# Discovering heuristics in a complex SAT solver with large language models

**DOI:** 10.1038/s41467-026-74949-2

**Published:** 2026-07-17

**Authors:** Yiwen Sun, Furong Ye, Zhihan Chen, Ke Wei, Shaowei Cai

**Affiliations:** 1 School of Data Science, Fudan University, Shanghai, China; 2 Key Laboratory of System Software, Institute of Software, Chinese Academy of Sciences, Beijing, China; 3 SeedMath Technology Limited, Beijing, China

**Keywords:** Computer science, Software

## Abstract

The Satisfiability problem (SAT) is fundamental in computational complexity theory and has a wide range of industrial applications. Optimizing modern SAT solvers in real-world settings is quite challenging due to their intricate architectures. While automatic configuration frameworks have been developed, they rely on manually constrained search spaces. Here we develop AutoModSAT, a framework that uses large language models (LLMs) to automatically optimize SAT solvers. AutoModSAT combines an LLM-compatible modular solver design, unsupervised prompt optimization to diversify generated functions, and an efficient search procedure based on presearch strategy and a (1 + *λ*) evolutionary algorithm. Extensive experiments across a wide range of datasets demonstrate that AutoModSAT achieves 40% performance improvement over the baseline solver and 30% improvement over the state-of-the-art solvers. Moreover, AutoModSAT also attains a notable speedup compared to the parameter-tuned alternatives of the state-of-the-art solvers over most of the test datasets. These results demonstrate the potential of LLM-guided heuristic discovery for optimizing complex SAT solvers.

## Abstract (web summary)

This study is on AI-driven SAT solver optimization. Here, authors developed AutoModSAT, an LLM-based framework for automatic heuristic discovery and solver optimization, achieving substantial performance gains over baseline and state-of-theart SAT solvers across diverse benchmarks.

## Introduction

The Satisfiability problem (SAT), a fundamental question in logic and computer science, asks whether there exists an assignment to variables in a given Boolean formula to make it true. As the first proven NP-complete problem^1^, SAT holds immense theoretical significance, and serves as a cornerstone in the computational complexity theory. Efficient solutions to SAT imply efficient solutions for all the problems in the NP class. Modern SAT solvers, driven by cutting-edge heuristics, are important for industrial applications, such as in semiconductor manufacturing, cybersecurity, automated reasoning and mission-critical software development^2,3^.

After decades of development, modern SAT solvers are equipped with complex heuristics, and industry users need to devote massive efforts to customize different SAT solvers for different real-world applications, which requires labor-intensive modifications by experts. For example, the Electronic Design Automation (EDA) task requires SAT solvers to handle various complex problem instances^4^. To address this issue, hyperparameter optimization approaches, also referred to as algorithm configuration, have been developed for automated SAT solver improvement^5–7^ so that they can choose well-performing settings for the given problems. However, the algorithm configuration approaches often suffer from manually-defined search spaces and suboptimal performance improvements.

Large Language Models (LLMs), the recent advance in generative artificial intelligence (AI)^8–14^, open up avenues for overcoming the limitations of manually designed frameworks^15^. After DeepMind’s pioneering work on complex mathematical and bin-packing problems^16^, LLMs have demonstrated promising applications in automated algorithm design^17–23^. However, few studies have been successfully done on optimizing programs that are as complex as SAT solvers. Firstly, SAT solvers often employ carefully customized data structures to improve computational efficiency, but meanwhile present challenges for automated optimization^19^. In addition, state-of-the-art SAT solvers, such as Kissat^24^ and CaDiCal^25^, have evolved over decades and thus contain hundreds of thousands of tokens, much larger than the context length limit of LLMs.

To tackle the challenges mentioned above, we propose AutoModSAT, an LLM-driven framework for SAT-solver optimization that is capable of generating competitive heuristics tailored to specific problem instances and thus eliminates repetitive manual tunings across different application scenarios. By leveraging LLMs, AutoModSAT is able to discover competitive heuristics programs beyond the human-designed search space in traditional hyperparameter optimization methods. Furthermore, we develop a well-defined modularized architecture to overcome the token length limitations of LLMs when working on complex solvers. Experimental results show that AutoModSAT achieves better performance across multiple datasets compared to commonly used solvers such as Kissat and CaDiCaL. Overall, AutoModSAT also outperforms the traditional hyperparameter optimization techniques in terms of both solution quality and computational efficiency.

This paper is structured as follows. Firstly, we outline the key components of AutoModSAT, including a modularized SAT solver to optimize, automatic prompt optimization, the presearch strategy and heuristics discovery. Secondly, we evaluate the performance of AutoModSAT across diverse scenario datasets, demonstrating its good performance compared to current SOTA SAT solvers. Then we discuss the insights obtained from AutoModSAT, potential limitations and future research directions. Finally, we provide a detailed description of each component within AutoModSAT, elaborating on its design principles and implementation details.

## Results

### AutoModSAT: An automatic heuristic discovery framework for SAT solvers

**Fig. 1:**
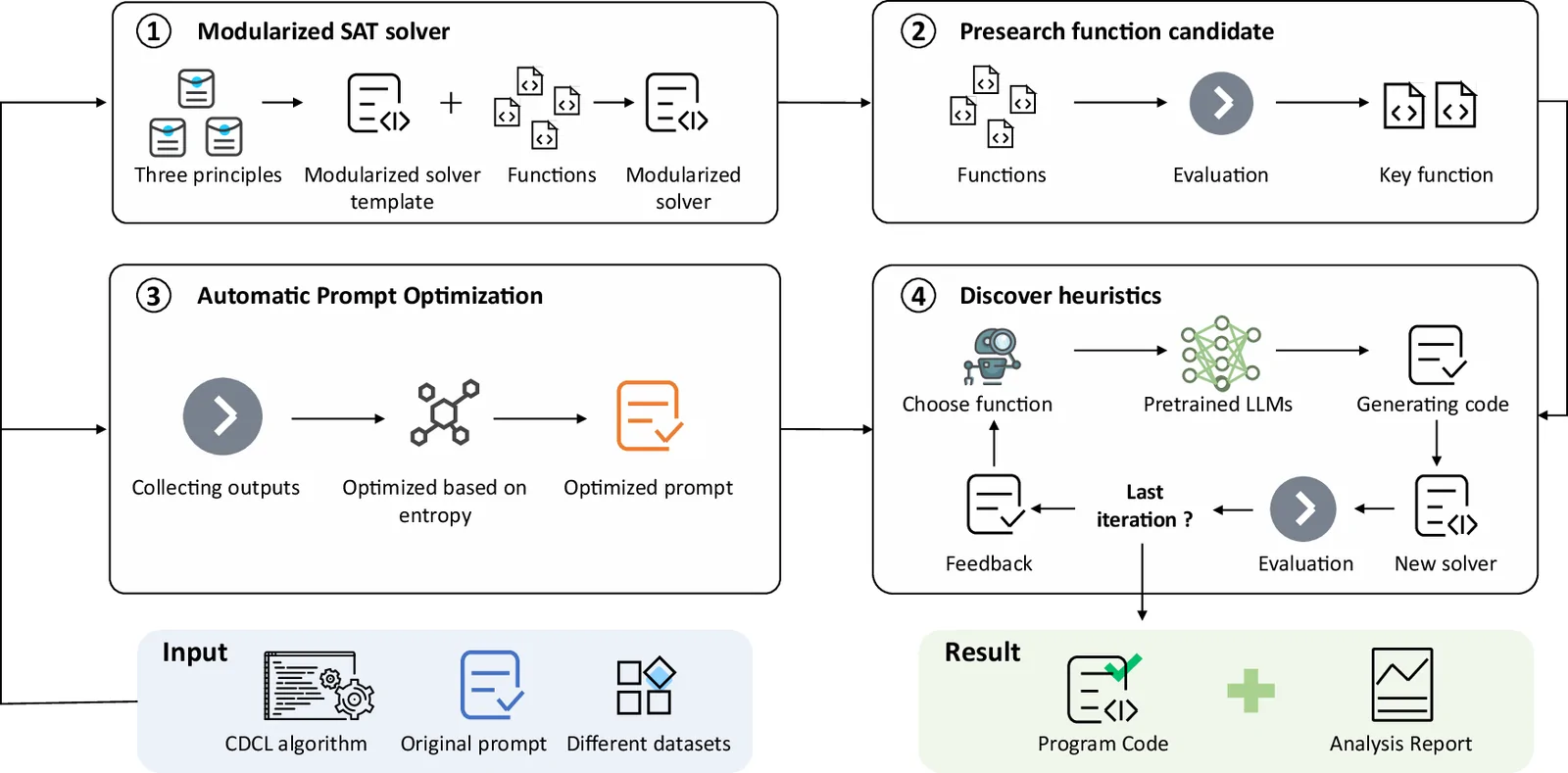
Overview of AutoModSAT. This figure displays the workflow of AutoModSAT, which distinguishes itself from existing LLMs methods for algorithm design on challenges in optimizing large-scale, structurally complex solvers. The whole process requires a basic CDCL algorithm, initial prompt, and datasets as input, then involves four core components: (1) a modularizing SAT solver (ModSAT) based on the CDCL algorithm; (2) a presearch strategy to identify function candidates for each dataset; (3) prompt optimization based on entropy; and (4) iterative heuristics discovery. After selecting the function to be optimized, we use LLMs to generate code, and evaluate it on a specific dataset if it can be executed successfully. More effective heuristics are then updated into the solver.

AutoModSAT is an LLM-driven framework designed to optimize complex SAT solvers. The whole framework is illustrated in Fig. 1, which comprises four core components.

- *Modularized SAT solver*. In order to meet the token length limit and compatibility of LLMs, we introduce ModSAT, a modularized SAT solver with well-designed functions. ModSAT is developed based on three principles, as detailed in the Method section. Instead of leveraging LLMs to generate a complete SAT solver from scratch, seven critical heuristics are defined in ModSAT which form a search space for LLMs to explore. Therefore, AutoModSAT can be expressed as a mapping from a set of heuristics to a solver, given by {*h*_1_, *h*_2_, …, *h*_7_} ↦ *A*, where each *h*_*i*_ represents a heuristic associated with a function and *A* is the resulting solver obtained by integrating these functions. The modularized solver enables LLMs to modify heuristics locally without disrupting components in other unrelated functions, facilitating reliable and efficient program generation.
- *Presearch strategy*. A desirable search space not only enables us to generate competitive solvers but can also improve our understanding of the LLM-based search process. An appropriate search space, tailored to the landscape of searching SAT solvers, has been identified through a presearch strategy involving preliminary candidate pruning.
- *Automatic prompt optimization*. Diverse prompts are necessary for LLMs to discover more effective heuristics. To this end, we adopt an unsupervised automatic prompt optimization method to increase the diversity of the prompt, which can enhance the performance of the discovered heuristics while reducing labor costs in prompt engineering.
- *Heuristics discovery*. After function candidates and prompts are obtained, we leverage three LLM-based agents, a coder, an evaluator, and a repairer to discover effective heuristics based on the (1 + *λ*) EA^26^ search strategy, yielding the final AutoModSAT solver. Extensive experiments across multiple benchmark datasets have demonstrated the superiority of AutoModSAT in efficiency and effectiveness, and provide a direction for future SAT solver development.

Overall, AutoModSAT is an LLM-driven optimization framework which can produce domain-specific SAT solvers, and thus can reduce both time and labor costs in real-world applications. It establishes the potential of utilizing LLMs to optimize large-scale solvers that have complex customized data structures and long-text codes. Based on the insights obtained from our practice in this work, it is possible that the proposed LLM-driven optimization framework may also be useful in optimizing other complex solvers.

### Discovery of effective heuristics

We selected 11 datasets to test the capability of AutoModSAT, consisting of 7 datasets from the SAT Competition 2023 and 2024^27,28^, 3 datasets generated by Picat^29^, and another dataset from a real industrial EDA scenario. For the selection of SAT Competition datasets, we filter out families with fewer than twenty instances as they are susceptible to overfitting during the iterative optimization process, and select datasets with substantial variations in average solving times in order to comprehensively evaluate a solver’s performance across diverse data types. As a result, a total of 7 families are tested: argumentation_2023, argumentation_2024, cryptography-ascon, register-allocation, social-golfer, hashtable-safety and hamiltonian. Another 3 datasets are manually generated by Picat^29^, including KnightTour, MineSweeper, and Zamkeller, which can formulate the constrained satisfied problems into Conjunctive Normal Form (CNF) formulas. The last dataset is from an industrial EDA scenario, which contains 50 industrial instances derived from combinational equivalence checking problems. The sources and characteristics of all datasets are summarized in Table 1, and more details are presented in Supplementary Section 4.1.

**Table 1:** Dataset information

dataset	total number of instances	source	variables mean	variables std	clauses mean	clauses std
cryptography-ascon	20	SC 2023	146,636	15,010	342,940	37,315
register-allocation	20	SC 2023	381	193	5,813	5,622
social-golfer	20	SC 2023	15,540	12,157	131,517	79,751
hashtable-safety	20	SC 2023	11,712,548	4,874,397	53,644,509	22,032,387
argumentation 2023	20	SC 2023	962	190	27,960	23,034
argumentation 2024	21	SC 2024	909	187	35,110	29,989
hamiltonian	40	SC 2024	511	60	4,062	533
MineSweeper	88	Picat	618,801	531,657	9,065,224	7,909,645
KnightTour	56	Picat	223,697	313,704	10,692,883	17,482,123
Zamkeller	80	Picat	24,592	22,190	310,804	335,102
EDA	50	Industrial	1,822	1,195	6,185	4,207

Datasets used in this paper are taken from the SAT Competition 2023, 2024 (SC 2023, SC 2024 for short), Picat (a language tool for problem generation), and the industrial EDA scenario. The size of each dataset and statistics on the number of variables and clauses are provided, which clearly show the diversity of the problem instances.

Apart from the classic CDCL-based SAT solver MiniSat^30^, we also compare with the SOTA SAT solvers Kissat^24^ and CaDiCal^25^, whose performance is fully optimized through hybrid heuristics and complex data structures. Even though Kissat and CaDiCal have demonstrated superior performance compared to ModSAT, the code complexity and opaque low-level data structures currently prevent effective modifications via LLMs. In addition to the original solvers, we also compare with their parameter-tuning versions in order to fully explore their abilities for different problem instances. More precisely, we employed SMAC3 (Sequential Model-based Algorithm Configuration)^31^ to optimize the parameters of the aforementioned SAT solvers. SMAC3 follows a Bayesian optimization framework and consists of three key steps: 1) define the configuration space, solver-specific parameters and their feasible ranges; 2) configure SMAC3 using a performance metric (e.g., PAR-2) and execute multiple optimization trials with instance-specific training datasets; 3) the best-found configuration is validated on the testing dataset. This approach systematically addresses parameter interdependencies while mitigating overfitting, leading to performance improvements over default settings. Note that PAR-2 is an evaluation metric that measures the performance of a SAT solver by averaging the runtime for solved instances while penalizing the unsolved instances with a double timeout penalty. Further details about PAR-2 are provided in the Supplementary Section 4.2. It should be emphasized that the focus in this work is the single-core SAT solver and that parallel SAT solving is out of scope, so all solvers are executed in the single-thread mode.

All experiments are conducted on five identical servers, each equipped with two AMD EPYC 7763 CPUs (64 physical cores per CPU) at a base frequency of 2.45 GHz. All SAT solvers are implemented in C++ with g++ 9.4.0, while the interfaces to LLMs and parameter-tuning methods are implemented in Python. The budget ${{{\mathscr{B}}}}$, referring to the number of times requesting new heuristics from LLMs or new parameters from SMAC3, is set to 50. We only report results obtained from DeepSeek-V3 in this paper due to its competitive performance and meanwhile the low cost in optimizing the SAT solvers, see Supplementary Section 4.4. Different time limits are used for the search process and the final evaluation. During heuristic search, the standard 5000 seconds timeout in SAT competition is excessively long, so we use the median solving time reported from the original solvers as the timeout. After obtaining the optimized solver, the original 5000 seconds timeout is used for the final evaluation. This approach significantly reduces the computational overhead while maintaining a meaningful assessment of performance. We adopt PAR-2 as the primary performance metric and additionally report the speedup between solvers, defined as: $\,{\mbox{speedup}}\,=\frac{{v}_{a}-{v}_{b}}{\max ({v}_{a},{v}_{b})}$, where *v*_*a*_ is the PAR-2 of method A, *v*_*b*_ is the PAR-2 of method B.

**Fig. 2:**
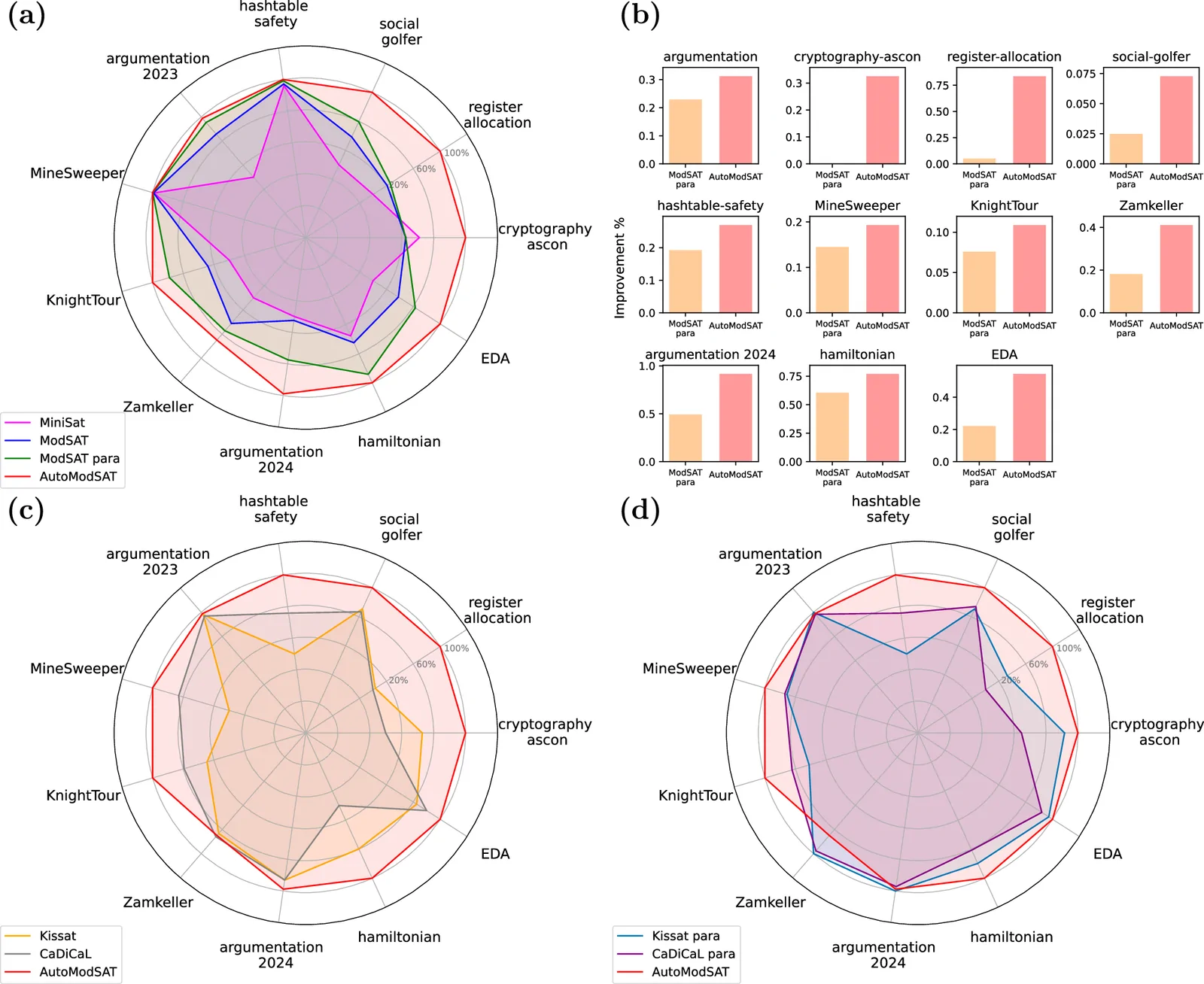
PAR-2 over Different Datasets. **a** This subfigure provides a visualization of PAR-2 in Table 2, which shows the improved performance of AutoModSAT over ModSAT. Here the plotted values are normalized for each dataset by $1-\frac{curr-min}{2*(max-min)}$, where *curr* is the PAR-2 corresponding to a solver, and *min* and *max* correspond to the minimum and maximum PAR-2 over all the tested solvers. A larger shadow area indicates better performance. **b** This subfigure provides a performance comparison between AutoModSAT and parameter-tuned ModSAT on improving the original ModSAT solver, where the vertical axis is the PAR-2 improvement ratio. **c** This subfigure compares the performance between AutoModSAT with Kissat and CaDiCaL. **d** This subfigure compares the performance of AutoModSAT with parameter tuning variants of Kissat and CaDiCaL.

The experimental results are presented in Fig. 2 and Table 2. It can be observed that AutoModSAT achieves over 40% performance improvement relative to ModSAT, and also outperforms its parameter-tuned version (denoted “ModSAT para”) with a 30% speedup. Furthermore, AutoModSAT exhibits substantial improvements over the SOTA solvers Kissat and CaDiCaL on each dataset, achieving an average performance gain exceeding 30%. Compared with the parameter-tuned Kissat and CaDiCaL (denoted “Kissat para” and “CaDiCaL para”), AutoModSAT achieves the shortest PAR-2 score while matching or exceeding the solved instance count in 8 out of the 11 datasets, including cryptography-ascon, register-allocation, social-golfer, hashtable-safety, hamiltonian, EDA, Minesweeper and KnightTour. Note that “Kissat para” achieves the best performance on Zamkeller, substantially better than AutoModSAT. The potential reason for the huge improvement from Kissat to “Kissat para” is investigated in Table 3.

**Table 2:** PAR-2 scores over different datasets

Solver	cryptography-ascon	register-allocation	social-golfer	hashtable-safety	argumentation 2023	argumentation 2024
MiniSat	193.62 ± 2.37 (20.00 ± 0.00)	8822.50 ± 15.73 (3.00 ± 0.00)	8175.59 ± 153.24 (4.20 ± 0.56)	91.81 ± 1.39 (20.00 ± 0.00)	8649.53 ± 5.89 (3.00 ± 0.00)	2887.18 ± 37.16 (17.00 ± 0.00)
ModSAT	207.76 ± 2.96 (20.00 ± 0.00)	7149.15 ± 143.40 (5.80 ± 0.30)	7882.76 ± 123.35 (4.90 ± 0.41)	83.44 ± 1.71 (20.00 ± 0.00)	4704.91 ± 68.00 (13.00 ± 0.00)	2738.42 ± 53.39 (17.00 ± 0.00)
ModSAT para	209.49 ± 3.93 (20.00 ± 0.00)	6878.99 ± 105.95 (6.30 ± 0.35)	7690.98 ± 116.70 (5.00 ± 0.34)	67.13 ± 0.87 (20.00 ± 0.00)	3650.53 ± 64.55 (14.00 ± 0.00)	1402.98 ± 25.15 (19.00 ± 0.00)
Kissat	191.19 ± 3.64 (20.00 ± 0.00)	8500.85 ± 250.66 (4.00 ± 0.34)	7564.10 ± 140.31 (5.10 ± 0.53)	456.85 ± 13.34 (20.00 ± 0.00)	3317.60 ± 79.87 (14.00 ± 0.00)	539.85 ± 16.14 (21.00 ± 0.00)
Kissat para	155.63 ± 2.86 (20.00 ± 0.00)	6303.36 ± 120.48 (8.20 ± 0.45)	7499.44 ± 161.03 (5.20 ± 0.56)	454.99 ± 11.62 (20.00 ± 0.00)	**3123.08 ± 68.63(14.00 ± 0.00)**	**169.86 ± 4.52(21.00 ± 0.00)**
CaDiCaL	230.20 ± 5.54 (20.00 ± 0.00)	8749.83 ± 4.29 (3.00 ± 0.00)	7516.27 ± 156.29 (5.20 ± 0.56)	251.69 ± 5.53 (20.00 ± 0.00)	3443.29 ± 15.14 (14.00 ± 0.00)	553.38 ± 16.40 (21.00 ± 0.00)
CaDiCaL para	203.03 ± 4.14 (20.00 ± 0.00)	8790.38 ± 243.37 (3.10 ± 0.23)	7452.61 ± 159.01 (5.20 ± 0.45)	251.09 ± 5.46 (20.00 ± 0.00)	3269.70 ± 95.18 (14.00 ± 0.00)	322.50 ± 8.44 (21.00 ± 0.00)
AutoModSAT	**140.80 ± 1.34(20.00 ± 0.00)**	**1178.31 ± 17.45(18.00 ± 0.00)**	**7253.67 ± 76.83(5.90 ± 0.23)**	**60.62 ± 0.65(20.00 ± 0.00)**	3228.13 ± 46.40 (14.00 ± 0.00)	232.53 ± 2.36 (21.00 ± 0.00)

Solver	hamiltonian	MineSweeper	KnightTour	Zamkeller	EDA
MiniSat	652.42 ± 9.56 (38.00 ± 0.00)	9.86 ± 0.16 (88.00 ± 0.00)	9126.73 ± 116.35 (5.20 ± 0.30)	5720.77 ± 114.48 (37.20 ± 0.74)	1102.31 ± 16.22 (45.00 ± 0.00)
ModSAT	581.26 ± 11.21 (38.00 ± 0.00)	9.18 ± 0.19 (88.00 ± 0.00)	8766.10 ± 148.88 (7.10 ± 0.41)	3472.66 ± 72.44 (54.10 ± 0.41)	829.92 ± 17.67 (46.00 ± 0.00)
ModSAT para	227.87 ± 4.35 (40.00 ± 0.00)	7.81 ± 0.13 (88.00 ± 0.00)	8175.10 ± 114.79 (11.10 ± 0.41)	2860.80 ± 61.65 (62.20 ± 0.45)	648.17 ± 11.29 (48.00 ± 0.00)
Kissat	456.84 ± 9.18 (39.00 ± 0.00)	144.89 ± 3.44 (88.00 ± 0.00)	8793.72 ± 257.04 (7.00 ± 0.67)	2218.62 ± 62.90 (65.10 ± 0.53)	626.82 ± 17.22 (48.00 ± 0.00)
Kissat para	294.64 ± 6.86 (39.00 ± 0.00)	47.53 ± 0.99 (88.00 ± 0.00)	8521.22 ± 178.18 (8.30 ± 0.48)	**487.88 ± 12.83(77.00 ± 0.00)**	418.82 ± 10.15 (49.00 ± 0.00)
CaDiCaL	931.46 ± 18.95 (38.00 ± 0.00)	54.36 ± 1.39 (88.00 ± 0.00)	8336.58 ± 217.55 (9.40 ± 0.60)	1930.20 ± 49.42 (67.10 ± 0.41)	523.22 ± 10.53 (49.00 ± 0.00)
CaDiCaL para	442.38 ± 9.26 (39.00 ± 0.00)	43.65 ± 1.01 (88.00 ± 0.00)	8321.33 ± 211.94 (10.20 ± 0.56)	725.78 ± 21.45 (75.10 ± 0.41)	489.28 ± 13.83 (49.00 ± 0.00)
AutoModSAT	**133.35 ± 1.31(40.00 ± 0.00)**	**7.32 ± 0.08(88.00 ± 0.00)**	**7829.74 ± 66.95(12.70 ± 0.35)**	2052.87 ± 27.06 (66.10 ± 0.23)	**375.28 ± 4.26(49.00 ± 0.00)**

Each cell reports the PAR-2 score with the 95% confidence interval, followed by the number of solved instances in brackets. Lower PAR-2 scores are better, and higher solved-instance counts are better. The results are calculated over ten runs with random seeds from 1 to 10. Confidence intervals are computed with the Student-*t* distribution (df = 9). Bold values indicate the best PAR-2 score and the corresponding solved-instance count for each dataset.

**Table 3:** Ablation studies of Kissat options for Zamkeller

Setting	Change from default	PAR-2
Kissat default	–	2229.90
Kissat para	(full tuned config)	486.75
One-factor ablation: KISSAT DEFAULT + {one option}		
Ablate-eliminate	eliminate=0	560.59
Ablate-phase	phase=0	1999.49
Ablate-probe	probe=0	2139.03
Ablate-reduceint	reduceint=1e4	1739.60
Ablate-rephaseint	rephaseint=1e4	1822.06
Ablate-restartmargin	restartmargin=25	1850.55
Ablate-simplify	simplify=0	495.96
Ablate-stable	stable=2	1874.50
Ablate-target	target=0	4138.99
Ablate-tier1	tier1=4	1829.36
Ablate-tier2	tier2=8	1873.41
Two-factor combination: KISSAT DEFAULT + {eliminate=0, simplify=0}		
Ablate-eliminate-simplify	eliminate=0, simplify=0	488.25

**One-factor ablation** starts from KISSAT DEFAULT and sets exactly one parameter to the tuned value. Two-factor evaluates the combination eliminate=0+simplify=0. The PAR-2 (lower is better) with 5000s timeout is reported.

We have also conducted the in-domain and cross-domain generalization tests on the solvers produced by AutoModSAT. The details and the results are presented in Supplementary Section 4.6. As an automation framework of domain-specific solvers, it is expected that the solvers should generalize well for test data in the same domain, while it will be difficult for the solvers to generalize across domains, which matches our observations from the experimental results.

**Fig. 3:**
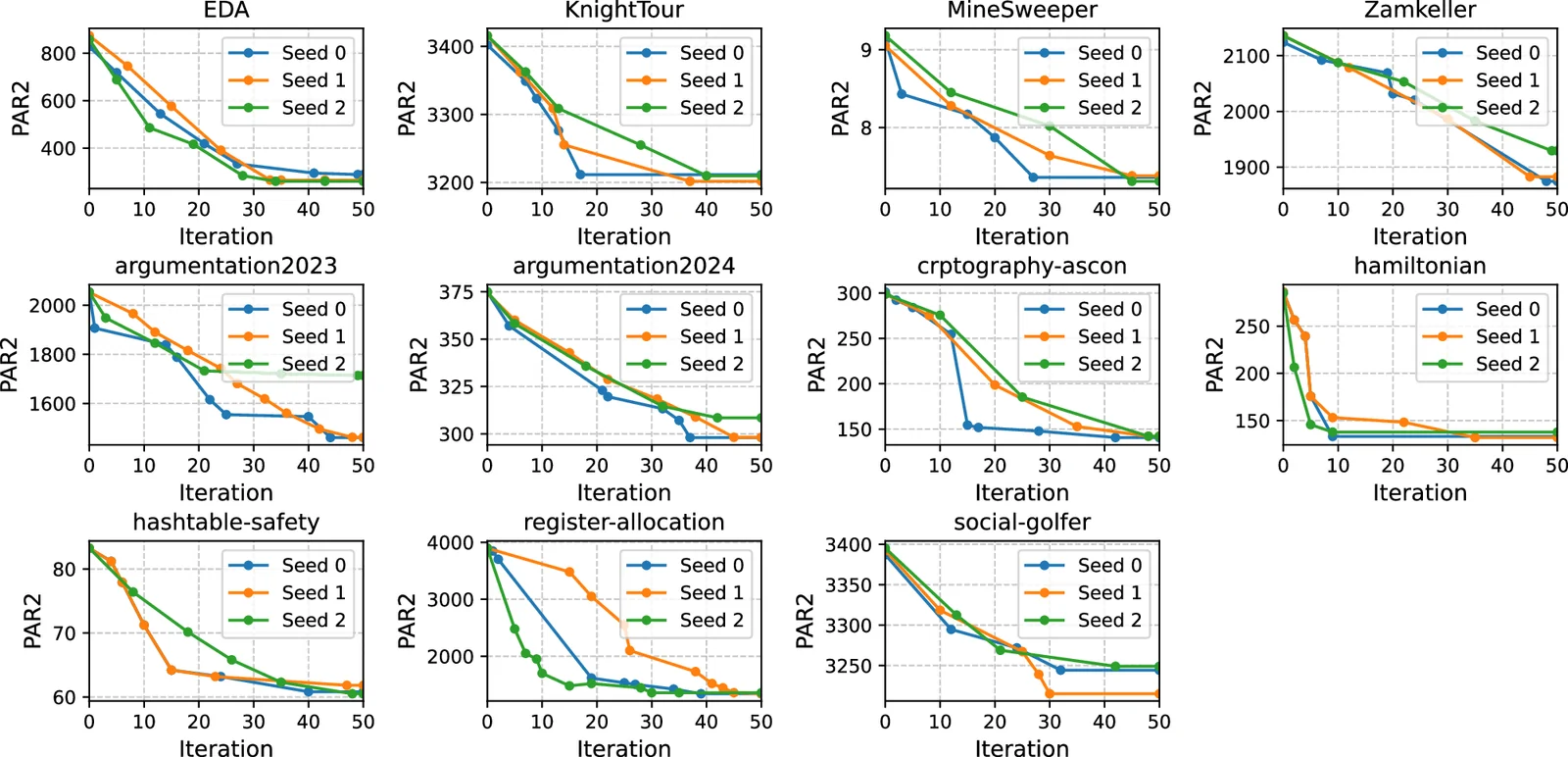
Iteration Process over Different Datasets. This figure shows the AutoModSAT optimization trajectory on each dataset for three different runs with different random seeds. The horizontal axis denotes the iteration count and the vertical is PAR-2.

In addition, we show the iteration process of the heuristic discovery in Fig. 3, where three experiments are conducted independently. The reported PAR-2 here reflects the search-phase setting (training subset and reduced training timeout) and is meant to visualize the optimization trend rather than to be compared with the final results in Table 2. It can be seen that AutoModSAT gradually produces the effective heuristics for every dataset.

Finally, we report the computational time (average runtime with 95% confidence intervals) required in the heuristic discovery process, which involves three LLM agents. The LLM coder requires 21.05  ± 0.55 seconds, the LLM evaluator requires 20.00  ± 0.91 seconds, and the LLM repairer requires 20.37  ± 0.51 seconds. The runtime is fairly balanced across agents, with each agent typically costing around 20 seconds. Therefore, one full iteration usually takes about 1–2 minutes (and can increase when repeated failures trigger additional repair cycles). More details, as well as a comparison across different LLMs, are provided in Supplementary Table 6.

### Investigating the Case on Zamkeller

To understand why “Kissat para” is superior on Zamkeller, we conduct two controlled ablation studies.

- **One-factor ablation**. Starting from default configuration of Kissat, we enable exactly one tuned parameter at a time, while keeping all other parameters at default values. This isolates the marginal effect of each parameter.
- **Two-factor combination**. From the one-factor results, we identify the two parameters with the largest positive impact on Zamkeller. We then evaluate the combination: Kissat default + {eliminate=0, simplify=0}, and compare it against “Kissat para”.

The PAR-2 scores for every setting is presented in Table 3. It is clear that eliminate=0 together with simplify=0 explain the majority of the performance gain of “Kissat para” over Kissat on Zamkeller. This suggests that aggressive preprocessing is detrimental on Zamkeller under the default Kissat configuration. In contrast, the current version of AutoModSAT only searches over 7 functions and does not control top-level preprocessing switches such as eliminate and simplify. Note that if we disable these two parameters manually, the performance of AutoModSAT can indeed be further improved on Zamkeller. However, it is still far from competitive compared with “Kissat para” since the baseline solver ModSAT is relatively weak on this dataset.

### Analysis of discovered heuristics

**Fig. 4:**
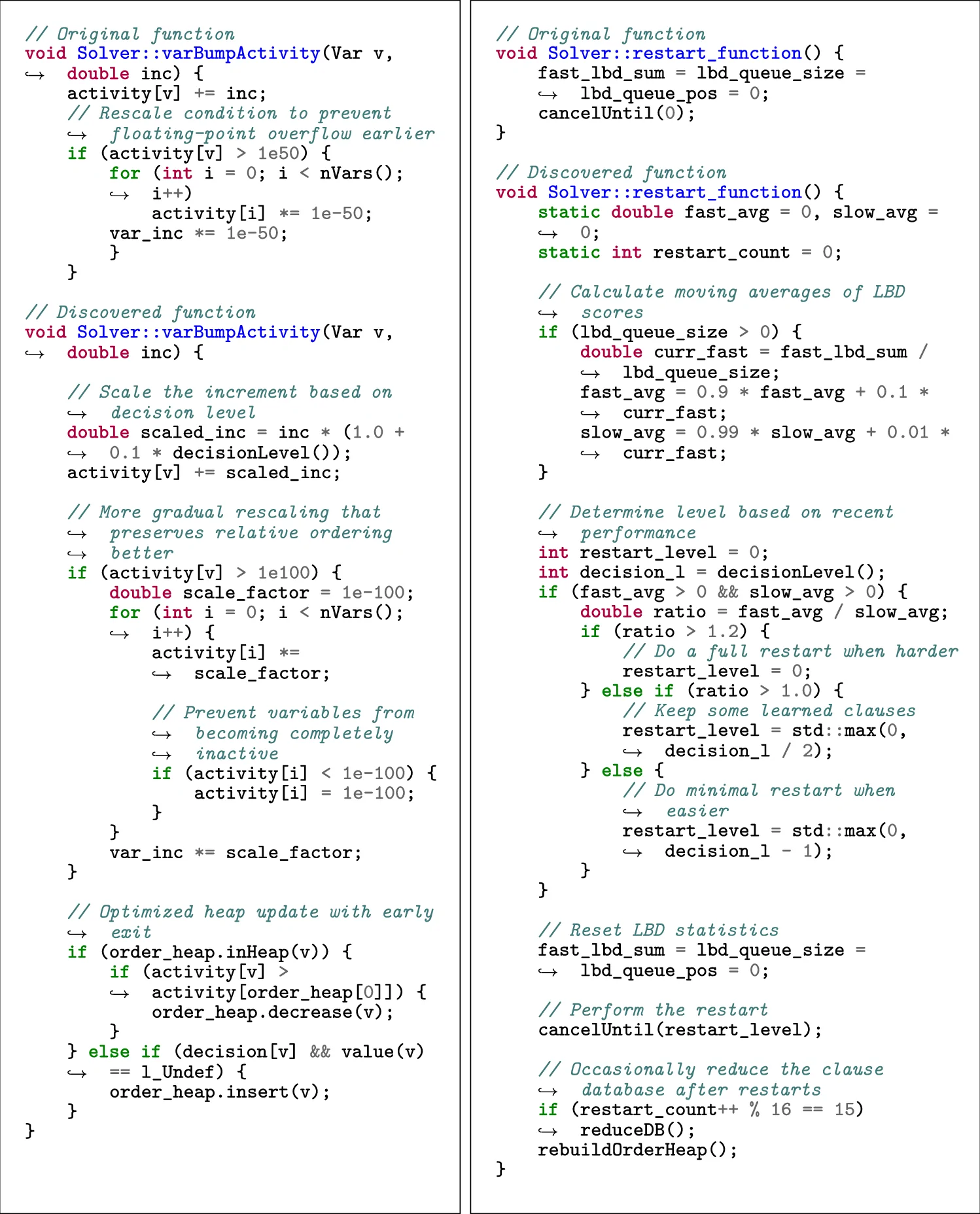
This figure shows two effective LLM-generated heuristic functions for SAT solvers and their original counterparts. (Left) The varBumpActivity function innovatively scales activity increments based on the solver’s current decision level. Both functions introduce unprecedented heuristic rules not previously proposed in existing solvers, demonstrating exceptional performance on EDA datasets. (Right) The restart function implements a dynamic restart strategy using moving averages of Literal Block Distance (LBD) scores.

AutoModSAT is capable of discovering effective heuristics, and we analyze two of them here: restart function and varBumpActivity as presented in Fig. 4. More examples are given in Supplementary Figs. 9-15. The heuristic varBumpActivity increases a variable’s activity score to prioritize it for future branching decisions. The generated varBumpActivity function introduces several key enhancements over the original one. For example, it scales the activity increments based on the current decision level of the solver (1.0 + 0.1*decisionLevel()), prioritizing variables involved in recent conflicts. This improves search efficiency by dynamically focusing heuristic guidance on newer decisions. Then, the rescaling mechanism uses a larger threshold (1*e*100 vs. 1*e*50) and a smaller scaling factor (1*e* − 100), which preserves relative activity differences more effectively while preventing floating-point overflow. Additionally, a minimum activity floor (1*e* − 100) ensures no variable becomes entirely inactive, maintaining heuristic relevance. Finally, the heap update logic is optimized to avoid redundant operations by only triggering a decrease when the variable’s activity surpasses the top elements of the heap. These changes reduce computational overhead while maintaining the ability to adaptively prioritize variables.

The heuristic restart_function can help the solver escape the local minimum. The generated restart_function introduces several key enhancements over the original implementation. For example, it implements a dynamic restart strategy using moving averages of Literal Block Distance (LBD) scores, maintaining both fast (90% historical weight) and slow (99% historical weight) averages. This enables adaptive decision-making: when recent conflicts become more complex (fast_avg/slow_avg ratio > 1.2), it performs a full restart; for moderate difficulty (ratio >1.0), it preserves some learned clauses through partial restart; and for easier conflicts, it minimizes backtracking. Moreover, it also introduces a periodic database reduction to help maintain memory efficiency. These improvements create a restart strategy that adapts to problem difficulty, and balances exploration and exploitation of learned clauses. It is worth noting that there are a few constants in the adaptive backtracking strategy introduced by LLMs. One should be careful about them since their values are simply the outputs of the LLMs and lack interpretability. In addition, there are static local variables in the LLM-generated restart_function. Even though this does not affect our experiments (one solver in a single-process), it would be problematic for parallel SAT solvers. In order to further address this issue, a potential solution is to instantiate the solver multiple times in a single process and record the standard statistics (e.g, conflicts, starts, decisions, propagations) of each instantiation and see whether they are identical. If not, we can trace back where the problem arises either manually or by LLMs.

## Discussion

The experimental results demonstrate that AutoModSAT achieves substantial performance improvements over both the baseline modularized solver (ModSAT) and existing state-of-the-art SAT solvers, and also attains a notable speedup compared to the parameter-tuned alternatives of the state-of-the-art solvers across multiple benchmark datasets. These gains demonstrate the hypothesis that LLMs can be leveraged to explore and optimize complex solvers.

A key innovation of AutoModSAT lies in the solver modularization principles. Traditional SAT solvers have deeply entangled components and large, legacy codebases that are difficult to optimize. By enforcing the three modularization principles and decoupled heuristics, it is easier for LLMs to reason about the solver’s behavior at a higher level of abstraction. Although this modularized solver is not as efficient as the well-established solvers such as CaDiCal or Kissat, it provides the necessary foundation for heuristic discovery, which ultimately leads to a more efficient new solver. The results also highlight the importance of automatic prompt optimization and presearch strategies. Without these mechanisms, the LLM-generated modifications tend to produce suboptimal heuristics. Entropy-based prompt optimization improves both the stability and diversity of the candidate heuristics, while the presearch stage effectively prunes the search space. Together, these components make the (1 + *λ*) EA optimization process more sample-efficient, enabling AutoModSAT to discover highly efficient solver variants within practical computational budgets. The ablation studies to investigate the effect of each component are provided in Supplementary Section 4.5.

Note that recent LLM-driven algorithm design methods (e.g., FunSearch, EoH, AlphaEvolve)^16,17,23^ show that LLMs can be used as search tools over code or heuristics generation. AutoModSAT is related in spirit, but it focuses on a practical gap that is less emphasized in prior work: making LLM-based optimization work reliably for SAT solvers that are large, entangled, and hard to automatically optimize reliably. In contrast to the aforementioned LLM-based algorithm design methods, AutoModSAT does not attempt to generate the entire algorithm from scratch due to the complexity of the SAT solvers as well as the quest for high performance. Compared to recent LLM-based AutoSAT^19^, AutoModSAT achieves much superior performance by introducing several new components, such as an LLM-friendly modularized solver design, automatic prompt optimization to increase code-generation diversity, and a presearch strategy to narrow down the effective search space.

Although these comparisons highlight the advantages of AutoModSAT over prior LLM-based algorithm design and SAT-solver optimization methods, several limitations remain and suggest directions for future work. Firstly, the baseline modularized solver can still be further optimized to provide a stronger starting point. AutoModSAT is not uniformly best across all datasets. While on families where ModSAT is relatively weak (e.g., Zamkeller), the parameter tuning variants of Kissat and CaDiCaL are still superior. Note that it may also be possible to further improve the performance of AutoModSAT by introducing more heuristic functions. Secondly, we would like to emphasize that even though the LLMs are used to discover the heuristics automatically, the entire process is not fully automatic. For example, it requires a human-in-the-loop cycle where an expert prepares the base solver. Lastly, the feedback integration between the search evaluation and the code generation is still heuristic. It is likely that more principled reinforcement or gradient-based feedback loops could make the optimization process more data-efficient.

## Methods

### A brief introduction of SAT

The SAT problem is defined as follows. Let *V* = {*x*_1_, *x*_2_, …, *x*_*n*_} be a set of Boolean variables. A literal is either a variable *x* ∈ *V* or its negation  ¬ *x*. A clause is a disjunction of literals. A conjunctive normal form (CNF) formula *F* is a conjunction of clauses. The goal of SAT is to determine whether there exists an assignment of binary values (True or False) to the variables in *V* such that the value of *F* is True.

The naive approach for SAT is to exhaustively enumerate all possible assignments. A complete SAT solver implements the search in a systematic way, and determines if there exists at least one assignment that satisfies *F* (proving *F* is satisfiable). If no satisfying assignment exists, the solver conclusively proves *F* is unsatisfiable. Within this framework, the strategies for branching (choosing which variable and what value to assign next) become critically important. Key techniques to enhance search efficiency include restart strategies, rephase strategies, and activity-based heuristics (often implemented by bumping variable or clause activity scores).

Conflict-Driven Clause Learning (CDCL)^32^ is the most common approach for complete SAT algorithms, which dominates in modern high-performance SAT solvers. The core innovation is conflict analysis: when a conflict occurs, the solver analyzes it and derives a new clause that explains the inconsistency. These derived clauses, called learnt clauses, enable the solver to prune large portions of the search space, significantly improving efficiency. However, an excessive number of learnt clauses can degrade the unit propagation speed and consume excessive memory. Consequently, identifying high-quality learnt clauses and reducing their quantity are also essential for maintaining solver performance. Therefore, achieving competitive performance in modern SAT solvers requires not only the theoretical advances, but also a series of engineering innovations, including efficient data structures and heuristic policies to realize these ideas effectively.

#### Algorithm 1

**ModSAT** 

### ModSAT: A Modularized SAT solver

The current architecture of complex SAT solvers has extensive functions, often resulting in poor modularity. This hinders the direct application of LLMs to solver optimization. Therefore, we develop a modularized solver, ModSAT (see Algorithm 1), by augmenting MiniSat^30^ with rephasing heuristics and then modularizing it according to three principles that will be detailed later. ModSAT follows the basic CDCL framework^32^, which usually initiates with an empty set of partial assignments (line 2). The Unit Propagation (UP), also called Boolean Constraint Propagation, assigns values to variable in clauses which has only one variable. UP can always make a clause satisfied, and this operation will repeat until no more UP is possible (line 4). If no conflicts are detected in ${{{\mathscr{X}}}}$ during the Decision Detection phase, the algorithm will select a variable to assign a value (line 19). This Make Decision step usually contains a few heuristics. When conflicts are detected (line 5), Analyze Conflict will identify the conflicted clauses (line 9), and a newly learnt clause will be derived based on the clause (i.e., the current partial assignment) of conflicts (line 10). Afterwards, the algorithm backtracks to an earlier decision level (lines 12-13). Once all variables are assigned and no conflict is detected, the algorithm obtains a satisfied assignment (line 28); otherwise, detecting conflicts at the decision level 0 indicates that the given CNF is unsatisfiable (line 7).

There are several important heuristics in ModSAT. For instance, *reduce* heuristics identify and remove the learnt clauses by controlling the size of the tracking list. In addition, *bump var heuristics* are usually incorporated in the Analyze Conflict step, which affect the choice of variables in the Make Decision function. While the order of choosing variables can determine the search path of branching, *rephase* heuristics can control the polarity in variables to be selected. Also, *restart* heuristics may abandon the current search path, allowing algorithms to explore possibly easier search regions. In particular, we have defined seven functions which are independently implemented: **restart function**, which manages restart heuristics; **restart condition**, which determines when to execute restart; **reduce condition**, which determines when to reduce; **rephase function**, which manages rephase heuristics; **rephase condition**, which determines when to rephase; **bump var activity**, which governs the order of variables selection in Make Decision; and **bump cla activity** that govern the order of clauses being removed during reduce. These functions collectively improve the solver’s ability to handle large and complex SAT instances by balancing exploration, exploitation, and resource management. The combination of the conflict-driven learning, backtracking, and heuristic enhancements makes ModSAT a robust SAT solver.

### Three principles behind ModSAT

As noted earlier, the high complexity of modern SAT solvers makes them difficult for LLMs to optimize. For instance, modifying a single variable may require changes across multiple locations, hindering the generation of correct code. Additionally, to enable localized heuristic modifications without disrupting unrelated components, adequate error tolerance must be allowed, which requires comprehensive variable context sharing for the modularized solver. To address these challenges, we have formulated three guiding principles:

- maintain functions simple and focus;
- use class variables for shared information;
- proactively prevent bugs during heuristics discovery.

**Maintain functions simple and focus** means that the function optimized by LLMs should be simple and explicit, which is uncommon in complex solvers. Contemporary solvers, typically implemented in C/C++, often employ complex data structures coupled with poorly modularized functions. This structural deficiency can mislead LLMs on variable scopes, so erroneous codes may be generated when optimizing the solvers.

**Use class variables for shared information** means that class member variables are more friendly than global variables for LLMs to access. Although functions typically utilize only a few variables in the original solver implementations, LLMs tend to coordinate with additional variables to strengthen the diversity of heuristics. Therefore, instead of providing the information on all possible variables in the prompt to LLMs, only relevant class member variables are provided as contextual information, which allows them to autonomously determine which variables to utilize.

**Proactively prevent bugs during heuristics discovery** means that we should fix the bugs written by LLMs proactively, so that the solver can compile correctly with the same heuristics. Many compilation errors admit straightforward resolutions, particularly those arising from common issues such as missing inclusions or variable scope misinterpretations. Fixing the bug proactively is helpful for LLMs to generate more diverse and correct codes.

### Presearch strategy

The goal of the presearch strategy is to prune low-value function candidates before the heuristic search. Previous work^19^ usually utilizes strategies such as evolutionary algorithms (EA) and greedy hill climber to optimize all the candidate functions, which becomes difficult to scale as the number of functions grows. For instance, in optimizing heuristic functions as a pseudo-Boolean optimization problem (e.g., LLMs provide merely two candidates for each function) with sequential dependency, EA requires approximately 0.54*n*^2^ (*n* is the number of functions need to be optimized) trials of generating new heuristics functions as illustrated in recent empirical studies^33,34^. However, the number of candidates for each function that LLMs can provide is more than two in practice, and the performance contribution of each function may depend on complex dependencies. These facts indicate that significant optimization time is required for achieving the optimal results^26,35^. Since evaluating a SAT solver is a time-consuming task with a timeout bound of 5, 000 seconds, searching for the optimal combination of SAT heuristic functions is computationally prohibitive.

To address this issue, we propose a presearch strategy to mitigate the combinatorial explosion: prune function candidates through small-scale preliminary tests (single-function evaluation on compact datasets), and then execute (1 + *λ*) EA (with *λ* = 1) search on the refined subset of functions. Our empirical analysis shows that, for a given dataset, some functions consistently degrade solver performance, which suggests a natural dimensionality-reduction strategy to accelerate the search for effective heuristics. This two-phase approach significantly reduces the iteration counts while preserving optimization effectiveness. Note that the (1 + *λ*) EA^26^ is an evolutionary algorithm where, in each generation, a single parent individual produces *λ* offspring through mutation, and the fittest individual among the parent and all offspring is selected as the parent for the next generation, ensuring elitist selection.

More precisely, we construct a representative subset comprising 50% of the problem instances from the original dataset. On this compact subset, each candidate function’s standalone impact based on the PAR-2 metric (where lower values indicate better solver performance) is evaluated. Typically, this phase retains about four high-impact functions that consistently improve the PAR-2 score, and functions that degrade the PAR-2 score are identified and pruned. For each dataset, the pruned functions are not considered in the subsequent evolutionary search. In practice, this phase typically retains a small set of high-impact functions (e.g., four functions) that consistently show a positive or neutral effect on PAR-2 in these preliminary tests. Subsequently, we execute a (1 + *λ*) EA on the full dataset using this refined function set in the following heuristics discovery process. The convergence behavior of AutoModSAT with and without the presearch (see Supplementary Fig. 8) show that the presearch can improve the computational efficiency of AutoModSAT.

### Prompt template and optimization

For clarity, we first introduce the basic prompt template, then explain the motivation for prompt optimization, and finally describe the optimization procedure. We begin with the basic prompt template for the LLM agents, which follows the instructions in OpenAI framework docs^36^ and has the following format:

- Define the **Role** of an agent as a solver expert who needs to assess and improve the heuristics function in the SAT solver.
- Clearly state the **Goal**, such as providing optimization suggestions, writing code, or feedback.
- Enhance the agents’ capabilities by inserting optional **Tips** that guide them to avoid common mistakes during code generation. Additionally, through this flexible interface, agents can effectively utilize external codes and results and can be instructed to specify the types of modification directions such as changing parameters, modifying heuristics, or adding new heuristics.
- Key code of SAT solver is appended at the end of each prompt to ensure all agents are in the same context. Note that the key code includes member variables in the cpp class of the solver (LLMs may utilize), along with the main loop function, and all corresponding functions LLMs may need to understand (e.g., such as the functions which would be called by the target optimized function).

The basic prompt template only provides a unified interface for different agents, yet identifying effective prompts remains challenging in practice^37^. Traditional approaches require extensive trial-and-error by experts to identify effective prompts, a process further complicated by the dynamic nature of optimal prompts as LLMs evolve. While conventional prompt optimization leverages supervised learning when labels can be obtained easily^38–40^, our task faces two critical constraints: prohibitive execution time (5000 seconds timeout per instance), and the absence of definitive labels due to the performance variance across different datasets.

To address these issues, an unsupervised method that utilizes Shannon entropy as an evaluation metric is adopted for automatic prompt optimization here. This metric quantifies the population diversity at each time step based on clusters of all the individuals. Firstly, we employ the CodeT5+ embedding model^41^ to generate embeddings for the synthesized code, which is preprocessed according to the Google C++ style guide^42^ (e.g., removing comments and ensuring uniform indentation). The resulting embeddings form the set **X**, where each code sample *x* ∈ **X** is represented by an *m*-dimensional vector. Then, we use an unsupervised learning method to cluster the *N* distinct code embeddings generated by the same prompt. Specifically, we apply the K-Means++^43^ to partition the embeddings into *K* clusters. More precisely, given the embeddings ${{{\bf{X}}}}=\{{x}_{1},\ldots,{x}_{N}\}\subset {{\mathbb{R}}}^{m}$, K-Means++ consists of the following three steps:

1. Initialize centroids: Select the first centroid *c*_1_ uniformly at random from **X**. For each subsequent centroid *c*_*j*_ (*j* = 2, …, *K*), choose it as *x* ∈ **X** with probability proportional to $\min_{k=1}^{j-1}|x-c_k|^{2};$
2. Assign clusters: Assign each embedding *x*_*i*_ to the nearest centroid *c*_*j*_ using Euclidean distance.
3. Update centroids: Recompute the centroids as the mean of all embeddings in each cluster, and then repeat step (2).

After the clustering is completed, we calculate the probability distribution of each cluster, denoted ${p}_{i}=\frac{| {C}_{i}| }{N}$ (*i* = 1, ⋯ *K*), where ∣*C*_*i*_∣ is the number of individuals of the *i*-th cluster. Then the entropy, *H*(*X*) is given by $H(X)=-{\sum }_{i=1}^{K}{p}_{i}\log ({p}_{i})$. This criterion is able to eliminate the dependency on explicit labels while maintaining adaptability to the evolution of LLMs, particularly suitable for compute-intensive optimization tasks with ambiguous success criteria.

Based on the Shannon entropy metric, the three components in the prompt templates can be optimized by LLMs. In each iteration, we randomly select a component to optimize by LLMs, compute the correctness and diversity of the generated codes, and update the component in prompt with better performance in both correctness and diversity. This loop will iterate until the stopping criteria is met, see Supplementary Algorithm 4 for details.

### Heuristics discovery

**Fig. 5:**
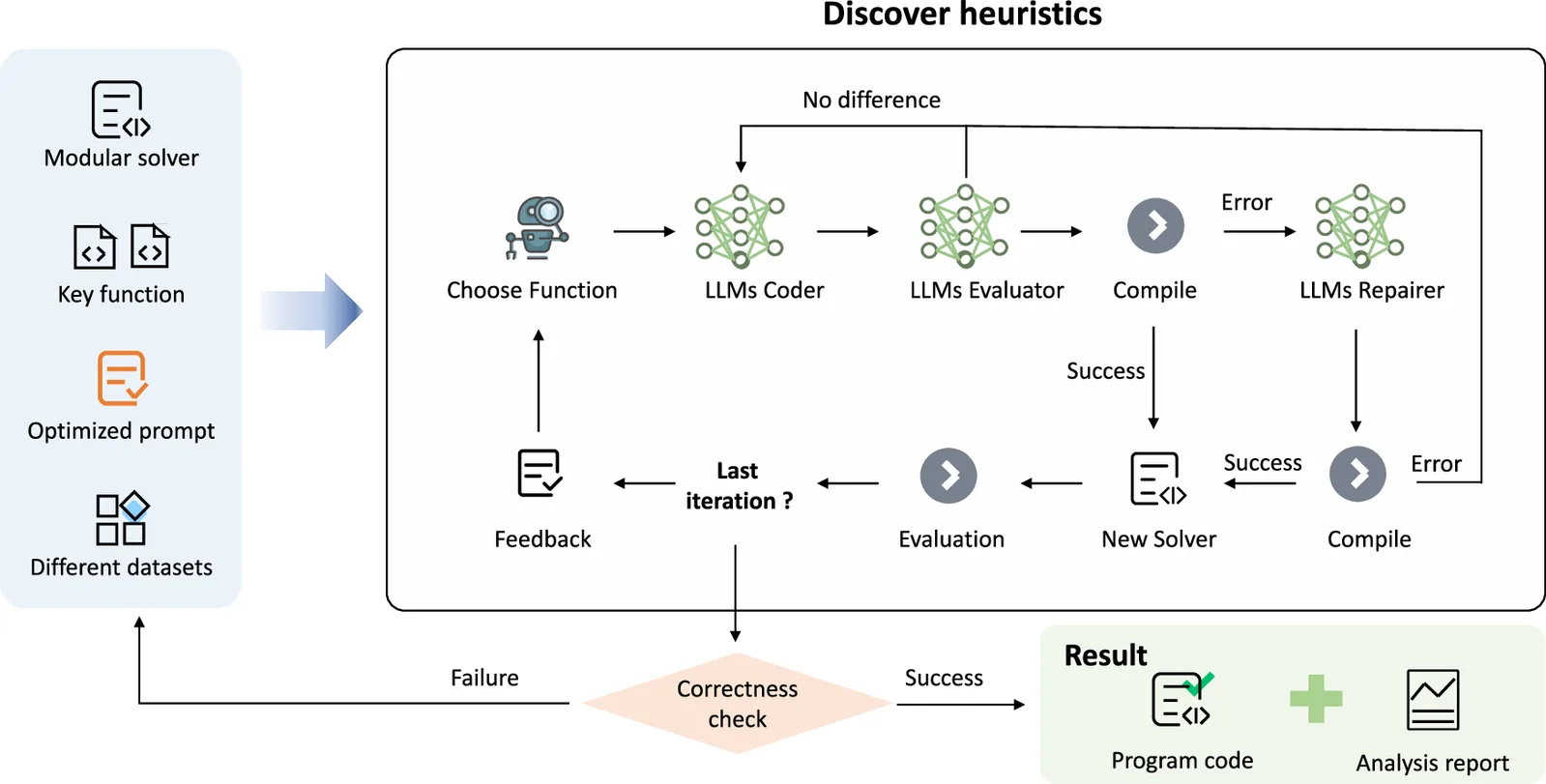
Heuristic discovery. This procedure iteratively implements LLMs agents to generate new heuristics. Heuristics that passed a synonymous check are evaluated on the datasets and higher-performing heuristics are integrated back into the new solver, while failures trigger automatic repair. The loop continues until the iteration budget is exhausted.

After obtaining the optimized prompt, the modularized SAT solver, and the presearch function candidates, AutoModSAT starts to discover heuristics in different datasets. This phase involves three LLM-based agents, including LLMs coder to generate heuristics, LLMs evaluator to filter out semantically equivalent code and avoid redundant search iterations, and LLMs repairer to correct common errors from LLM-generated code. See Fig. 5 for an illustration of this process.

Within each iteration of this process, a specific function is selected from the effective candidate space. The LLMs coder, whose prompt has been optimized in the automatic prompt optimization stage, is then called to generate code. Since we have observed that the LLMs coder may generate code that is syntactically different but functionally synonymous to the original implementation, the generated code is then evaluated by the LLMs evaluator. The code that passes the synonymous evaluation will be executed immediately against a predefined dataset. Successful execution triggers a performance evaluation phase to assess the heuristic’s efficacy.

If the code execution fails, the code along with the error output are sent back to LLMs repairer. LLMs repairer utilizes this feedback to generate revised code and attempt to repair the bug. However, if the repair attempt fails, then the current iteration for the selected function candidate is terminated and the process restarts with a new iteration, possibly selecting a different function candidate to optimize. Moreover, when the code execution succeeds, it will be evaluated and compared with existing heuristics. If the new heuristic demonstrates a superior performance, it is dynamically integrated into the modularized solver to enhance its capabilities.

Afterwards, we additionally verify the correctness of the discovered solver. For SAT instances, the solver outputs a satisfying assignment, which is checked against the original CNF formula. For UNSAT instances, the solver outputs a DRAT UNSAT proof that is verified using drat-trim^44^. Any solver candidate that fails this correctness check would be discarded. More details about the correctness verification can be found in Supplementary Section 4.7. In summary, the entire process consists of candidate selection, LLM-driven code generation, execution validation, bug repair, performance evaluation, and solver update, followed by the correctness verification. This structured framework facilitates systematic and adaptive code generation, and can successively refine the solver’s heuristic search space through successive iterations.

## Supplementary information

Supplementary Information

Transparent Peer Review file

## Data Availability

The datasets used in this study are available from the following sources. The benchmark instances from the SAT Competition 2023 and 2024 are publicly available at the official competition website: https://satcompetition.github.io/. The datasets generated by the Picat solver are available in the Zenodo repository: 10.5281/zenodo.17856423. The proprietary dataset from real industrial EDA scenarios is also included in the same Zenodo repository: 10.5281/zenodo.17856423.

## Code availability

The source code for AutoModSAT has been deposited in the Zenodo repository under the MIT License and is publicly available at: 10.5281/zenodo.17856395.
