# Supplementary material for: Discovering heuristics in a complex SAT solver with large language models
Source: Nat Commun. 2026 Jul 17;17:9008. doi: 10.1038/s41467-026-74949-2 (PMC13507263; doi:10.1038/s41467-026-74949-2)
Supplement: Supplementary file 1 [file 41467_2026_74949_MOESM1_ESM.pdf]

Supplementary Materials

Discovering heuristics in a complex SAT solver with large language models

Yiwen Sun, Furong Ye, Zhihan Chen, Ke Wei, Shaowei Cai

1 Additional Background on SAT

Let $V = \{x_1, x_2, \dots, x_n\}$ be a set of Boolean variables. Recall that *literal* is either a variable x or its negation $\neg x$, a *clause* is a disjunction of literals, while *conjunctive normal form* (CNF) formula $F = C_1 \wedge C_2 \wedge \cdots \wedge C_m$ is a conjunction of clauses. For simplicity we assume all clauses are non-tautological. In other words, no variable x occurs positively $(x \in C)$ and negatively $(\neg x \in C)$ in the same clause.

A (partial) mapping $\alpha : V \to \{0,1\}$ is called an *assignment*. If α maps all variables to a boolean value, it is termed a *complete* assignment; otherwise, it is referred to as a *partial* assignment. The value of a variable x under an assignment α is denoted as $\alpha[x]$. An assignment α satisfies a clause if at least one literal evaluates to true under α, and satisfies a CNF formula if it satisfies all its clauses. A CNF formula F is satisfiable if there is at least one satisfying assignment. The empty clause $\perp$ is always unsatisfiable and represents a *conflict*. SAT is the problem of deciding whether a given CNF formula is satisfiable.

To solve the SAT problem, a straightforward approach that is implemented in a complete SAT solver is to conduct exhaustively search over all possible truth assignments to the variables in V to determine if at least one satisfies F (proving F is satisfiable). If no satisfying assignment exists, the solver conclusively proves F is unsatisfiable. Within this framework, the strategies for branching (for choosing which variable to assign next and what value to assign it) are crucial. Key techniques to enhance the search efficiency include restart strategies, restart strategies, and activity-based heuristics (often implemented via the bumping variable or clause activity scores).

1.1 CDCL solver

Conflict-Driven Clause Learning (CDCL)[1] is the most common approach and plays a dominant role in modern high-performance SAT solvers, see Supplementary Algorithm 1 for a basic CDCL framework. The core idea is conflict analysis: when a conflict occurs, the solver analyzes it and derives a new clause that explains the inconsistency. These derived clauses, called learned clauses, enable the solver to prune large portions of the search space, which significantly improve the computational efficiency. However, an excessive number of learnt clauses can degrade unit propagation speed and thus consume excessive memory. Consequently, identifying high-quality learned clauses and reducing their quantity are essential for maintaining a solver's performance.

Specifically, CDCL solvers operate on a propagation and learning mechanism, complemented by decision heuristics. The implementation of propagation and learning is a standard practice and overall similar in different solvers. In contrast, the decision policies (for variable selection) and restart policies (for search restarts) which are crucial for performance vary significantly. Due to the need for manual design and the absence of rigorous mathematical proofs for their optimality, these policies are referred to as important heuristics.

There is a long history of research on these heuristics in SAT solvers, among which branching heuristics play a crucial role and continue to impact the performance of CDCL solvers. For example, Variable State Independent Decaying Sum (VSIDS)[2] is a family of branching heuristics that seek to assign a value to the most promising variable in the Make Decision phase. Another important branching heuristic is Learning-Rate Branching (LRB)[3], which frames branching as an optimization problem that picks a variable to maximize a metric called learning rate.

Restart heuristics are also essential for enhancing the performance of CDCL solvers. It allows the solver to abandon the current search path and backtrack to a specific decision level. In this process, the learnt clauses are usually maintained for the next search. Fast restart[4] is a widely used method, and Luby restarts[5] is also heavily used because they represent a prior optimal strategy. However, most implementations are now switched to Glucose-style restarts[6], which are widely used in the SAT Competition. For more details, see the extensive overview given by Armin Biere[7].

Rephase is another technique in CDCL solvers. The primary objective is to reset or adjust the current partial assignment, thereby enabling the solver to explore a diverse search space. PrecoSAT and PicoSAT[8] utilize a Jeroslow-Wang score[9], computed from either all clauses or only irredundant clauses, with rephasing intervals scheduled according to a Luby sequence.

Supplementary Algorithm 1: CDCL Framework

Input: A CNF F of SAT instance;
Initialization: decision level $d \leftarrow 0$, current assignment of variables $\mathscr{X} \leftarrow \emptyset$;
while *True* **do**
  $\mathscr{X} \leftarrow$ **Unit Propagation**$(F, \mathscr{X})$;
  if *Conflicts are detected in* $\mathscr{X}$ **then**
    if $d == 0$ **then**
      return UNSAT
    else
      $\mathscr{C}_{conflict}, d_{backtrack} \leftarrow$ **Analyze Conflict**$(F, \mathscr{X})$;
      $C_{learned} \leftarrow$ **Learn Clause**$(\mathscr{C}_{conflict}, \mathscr{X})$;
      $F \leftarrow F \wedge C_{learned}$;
      $\mathscr{X} \leftarrow$ **Backtrack**$(\mathscr{X}, d_{backtrack})$;
      $d \leftarrow d_{backtrack}$;
  else
    if *All variables are assigned with a value* **then**
      return SAT
    else
      $d \leftarrow d + 1$;
      $\mathscr{X} \leftarrow$ **Make Decision**$(\mathscr{X})$

StrangeNight[10] employs a strategy of flipping values with a certain probability that depends on the depth of the assignment. The motivation is to avoid the heavy-tail phenomenon. These rephasing heuristics have been used and compared in the SAT solver Riss[11].

2 Illustration of Three Principles behind ModSAT

As already mentioned in the paper, there are three principles when developing ModSAT to ensure it is LLM-friendly:

- **Maintain functions simple and focus.** The function optimized by LLMs should be simple and explicit, unlike common implementations in complex solvers.
- **Utilize class variables for shared information.** Local variables should be declared as class member variables to give LLMs access to them.
- **Proactively prevent bugs during heuristics discovery.** The bugs written by LLMs should be fixed proactively, so that the solver could compile correctly with the same heuristics, which helps LLMs to generate more diverse correct codes.

Supplementary Figure 2 demonstrates the application of the principle *Maintain functions simple and focus*, with the original function given in Supplementary Figure 1. Instead of modifying the whole search function by LLMs, we modularize it into three distinct functions by isolating the components that significantly impact the performance and are suitable for LLMs to modify. The decomposition also enables LLMs to focus on refining one kind of heuristic at a time, thereby enhancing the code generation capability.

For the principle *utilize class variables for shared information*, we move variables that may reside in local scope into class members to ensure that LLMs can access this shared information, see Supplementary Figure 3.

Supplementary Figure 4 and Supplementary Figure 5 provide two examples of how to *proactively prevent bugs during heuristics discovery*. The first one is to include some extra packages to prevent LLMs from implementing extra functions, while the second one is to overload common functions to prevent LLMs from incorrectly implementing simple functions due to a misunderstanding of data structures.

3 More on Presearch Strategy and Automatic Prompt Optimization

The detailed description of the presearch strategy can be found in Supplementary Algorithm 2 and Supplementary Algorithm 3. In addition, we also investigate the contribution of each function to the final results by calculating the number of times that

Maintain functions simple: Original function to modify

```
lbool Solver::search(int nof_conflicts){
    // if there is a conflict
    ...... ......
    // if there is no conflict
    if ((lbd_queue_size == 50 && 0.8 * fast_lbd_sum / lbd_queue_size > slow_lbd_sum /
    conflicts) || !withinBudget())
        restart_function();

    // Simplify the set of problem clauses:
    if (decisionLevel() == 0 && !simplify())
        return l_False;

    // Reduce the set of learnt clauses:
        if (learnts.size()-nAssigns() >= max_learnts)
                reduceDB();
    if (rephase_condition())
        rephase_function();

    Lit next = lit_Undef;
    while (decisionLevel() < assumptions.size()){
        // Perform user provided assumption:
        Lit p = assumptions[decisionLevel()];
        if (value(p) == l_True){
            // Dummy decision level:
            newDecisionLevel();
            }else if (value(p) == l_False){
                analyzeFinal(~p, conflict);
                return l_False;
            }else{
                next = p;
                break;
            }
        }

    if (next == lit_Undef){
        // New variable decision:
        decisions++;
        next = pickBranchLit();

        if (next == lit_Undef)
            // Model found:
            return l_True;
    }
    newDecisionLevel();
    uncheckedEnqueue(next);
}
```

Supplementary Figure 1. Illustration of principle *maintain functions simple and focus.*

a function contributes to the final performance improvement in each experiment, see Supplementary Figure 6, where each subfigure shows the results for one dataset. It can be observed that almost all the selected functions contribute substantially to the final performance.

The pseudocode for automatic prompt optimization is presented in Supplementary Algorithm 4. Examples of the original prompt and the one after optimization are provided in Supplementary Figure 7.

Maintain functions simple: modularized function to modify

```
// original function which has been modularized
lbool Solver::search(int nof_conflicts){
    ...... ......

    if (restart_condition())
        restart_function();
    if (reduce_condition())
        reduceDB();

    if (rephase_condition())
        rephase_function();

    ...... ......
}

// functions to modify
bool Solver::rephase_condition() {
    if (rephases >= rephase_limit)
        return true;
    else
        return false;
}

bool Solver::reduce_condition() {
    if (rephases >= rephase_limit)
        return true;
    else
        return false;
}

bool Solver::restart_condition(){
    if ((lbd_queue_size == 50 && 0.8 * fast_lbd_sum / lbd_queue_size > slow_lbd_sum /
    conflicts) || !withinBudget())
        return true;
    else
        return false;
}
```

Supplementary Figure 2. Illustration of principle *maintain functions simple and focus*.

Supplementary Algorithm 2: Presearch Strategy in AutoModSAT

1 **Input:** Datasets P, modularized SAT solver with seven functions $\{h_1, \ldots, h_7\}$, prompt template, baseline functions $\{b_1, \ldots, b_7\}$
2 $P_{\text{compact}} \leftarrow 50\%$ representative instances from P
3 $R \leftarrow \emptyset$; `// Retained function set`
4 **for** *each function* $h_i \in \{h_1, \ldots, h_7\}$ **do**
5     $A_{\text{test}} \leftarrow$ build solver replacing h_i with baseline b_i
6     $f_i, s_i \leftarrow$ evaluate(A_{test}, P_{compact}) ; `// Get PAR-2 metric`
7 $F \leftarrow$ sort($f_1, \ldots, f_7$) ; `// Sort PAR-2 metric in different functions`
8 Get top 4 function index R from F

Add class member variables

```
// LBD heuristics
int lbd_queue[500],   // circled queue saved the recent 500 LBDs.
    lbd_queue_size,   // The number of LBDs in this queue
    lbd_queue_pos;
double fast_lbd_sum, slow_lbd_sum;

// rephase heuristics
int rephases, rephase_limit, rephase_count, threshold;
double last_rephase_progress;

// restart heuristics
int curr_restarts;
double last_restart_progress;
```

Supplementary Figure 3. Illustration of principle *utilize class variables for shared information.*

Prevent bugs: add more packages

```
#include <math.h>
#include <unordered_set>
#include <algorithm>
using namespace std;
```

Supplementary Figure 4. Illustration of principle *proactively prevent bugs during heuristics discovery.*

Supplementary Algorithm 3: Heuristics Discovery Strategy in AutoModSAT

Input: Datasets P, modularized SAT solver with pruned four functions $\{h_1, \ldots, h_4\}$, prompt template, baseline functions $\{b_1, \ldots, b_4\}$
$A \leftarrow$ solver with functions: $(\forall i \in R : h_i) \cup (\forall i \notin R : b_i)$
$f^* \leftarrow$ evaluate(A, P) ; `// Full dataset evaluation`
evalBudget $\leftarrow 50$; `// Maximum evaluations`
while *evalBudget* > 0 **do**
    $M \leftarrow \emptyset$
    $\ell \sim \mathrm{Bin}(|R|, \frac{1}{|R|})$; `// Sample modification count based on (1+λ) EA`
    chosen ℓ distinct values $M \leftarrow \{m_0, \ldots, m_\ell\}$ from R uniformly at random;
    Generate new functions $\{h'_m\}_{m \in M}$ via LLMs using A and M
    $A' \leftarrow$ update A with $\{h'_m\}_{m \in M}$
    $f(A') \leftarrow$ evaluate(A', P)
    if $f(A') \leq f^*$ **then**
        $A \leftarrow A'$
        $f^* \leftarrow f(A')$
    evalBudget $\leftarrow$ evalBudget $- 1$

4 Experimental details

4.1 Dataset description

Here we give more details about the datasets, including 7 datasets selected from SAT Competition 2023 and 2024, 3 generated ones by Picat, and another one from an industrial EDA scenario.

Prevent Bugs: overloading functions

```
#include <type_traits>

template <typename T1, typename T2>
typename std::common_type<T1, T2>::type max(T1 a, T2 b) {
    static_assert(std::is_integral<T1>::value && std::is_integral<T2>::value,
                  "max: Both types must be integers (int or long int)");
    return (a < b) ? b : a;
}

template <typename T1, typename T2>
typename std::common_type<T1, T2>::type min(T1 a, T2 b) {
    static_assert(std::is_integral<T1>::value && std::is_integral<T2>::value,
                  "min: Both types must be integers (int or long int)");
    return (a < b) ? a : b;
}

template <typename T1, typename T2>
typename std::common_type<T1, T2>::type max(T1 a, T2 b) {
    static_assert(std::is_floating_point<T1>::value && std::is_floating_point<T2>::value,
                  "max: Both types must be floating-point (float or double)");
    return (a < b) ? b : a;
}

template <typename T1, typename T2>
typename std::common_type<T1, T2>::type min(T1 a, T2 b) {
    static_assert(std::is_floating_point<T1>::value && std::is_floating_point<T2>::value,
                  "min: Both types must be floating-point (float or double)");
    return (a < b) ? a : b;
}
```

Supplementary Figure 5. Illustration of principle *proactively prevent bugs during heuristics discovery.*

- **Argumentation** involves finding acceptable sets of arguments in a directed graph where attacks between arguments are represented by edges.
- **Social Golfer** is a combinatorial problem that aims to assign golfers to groups over several weeks, ensuring no two golfers play in the same group more than once.
- **Hashtable Safety** focuses on verifying the correctness of operations in a hash table to avoid collisions and ensure the integrity of the structure.
- **Register Allocation** is a problem that arises in compiler optimization, where the goal is to assign a limited number of CPU registers to variables in a program.
- **Cryptography-Ascon** is a lightweight cryptographic algorithm challenge which focuses on implementing and verifying the Ascon cipher, a NIST-standardized authenticated encryption scheme, for resource-constrained IoT devices, balancing security against differential attacks with minimal computational overhead.
- **Hamiltonian** is a graph theory problem that involves determining whether a given graph contains a Hamiltonian cycle, a closed loop visiting each vertex exactly once, which is NP-complete and often applied to route optimization or circuit design verification.
- **MineSweeper** is derived from the classic MineSweeper game, where the objective is to determine the placement of hidden mines on a grid based on numerical clues.

Supplementary Algorithm 4: Automatic Prompt Optimization

Input: initial prompt template P, solver codebase S, max_iterations i, prompt optimized part $R = \{Role, Goal, Tips\}$

Output: optimized prompt template P^*

```
i ← 10                                                    // prompt optimization iterations
j ← 20                                        // number of code generation in each iteration
while i ≥ 0 do
    select prompt part r from R uniformly at random
    P′ ← refine_prompt(r, P)                             // LLMs refine the prompt template
    while j ≥ 0 do
        generated code c ← call_llm(current_prompt)
        compilation error e ← compile(c, S)
        if ¬e then
            C ← C ∪ c
        else
            execute_code(corrected_code)                                    // execute code

        end
        j ← j − 1
    end
    diversity d_i ← compute_code_diversity(C)
    success rate s_i ← compute_code_success(C)
    if d_i > d and s_i > threshold then
        d ← d_i
        P ← update_prompt(S, P′)
    else
    end
    i ← i − 1
end
```

- **KnightTour** aims to find a path for a knight on a chessboard that visits every square exactly once, with possible extensions to different board sizes and types.
- **Zamkeller** involves finding a permutation of integers from 1 to n that maximizes the number of differential alternations in subsequences divisible by integers from 1 to k, where $1 < k < n$.
- **EDA** involves formally proving whether two design specifications are functionally equivalent, which is one of the most essential techniques in Electronic Design Automation and digital IC design. It has a wide range of applications, such as functional equivalent logic removal, sequential equivalence checking, circuit-based method for symmetries detection, engineering change orders, among others.

For the generated instances using Picat[12], we adopt the settings in Chapters 2 and 3 of the book by Nengfa Zhou[12] and conduct grid sampling within a parameter space. Specifically, the parameter space $\Theta = \{\theta_1, \theta_2, \ldots\}, \theta_i^L \leq \theta_i \leq \theta_i^U$ is defined to ensure that three baseline solvers including MiniSat, CaDiCal and Kissat, can obtain a solution within proper time range, i.e., [1, 5000] seconds. Then we apply a space Θ' to generate the dataset by enlarging the upper bound of Θ, e.g., $\theta_i'^U = \theta_i'^U * 1.2$, such that we can test whether AutoModSAT can solve the instances where the baseline solvers cannot.

- **MineSweeper**
  Parameter Θ: $\{m, n, k, p\}$;
  Parameter Space: $[500, 1600] \times [400, 3200] \times [72689, 1572118] \times [0.32, 0.38]$;
  Notes: m, n represent the grid size of the Minesweeper game, k is the total number of mines, and p is the probability that a given cell contains a mine (range: 0.32 to 0.38).
- **KnightTour**
  Parameter Θ: $\{k\}$;
  Parameter Space: $[12, 75]$;

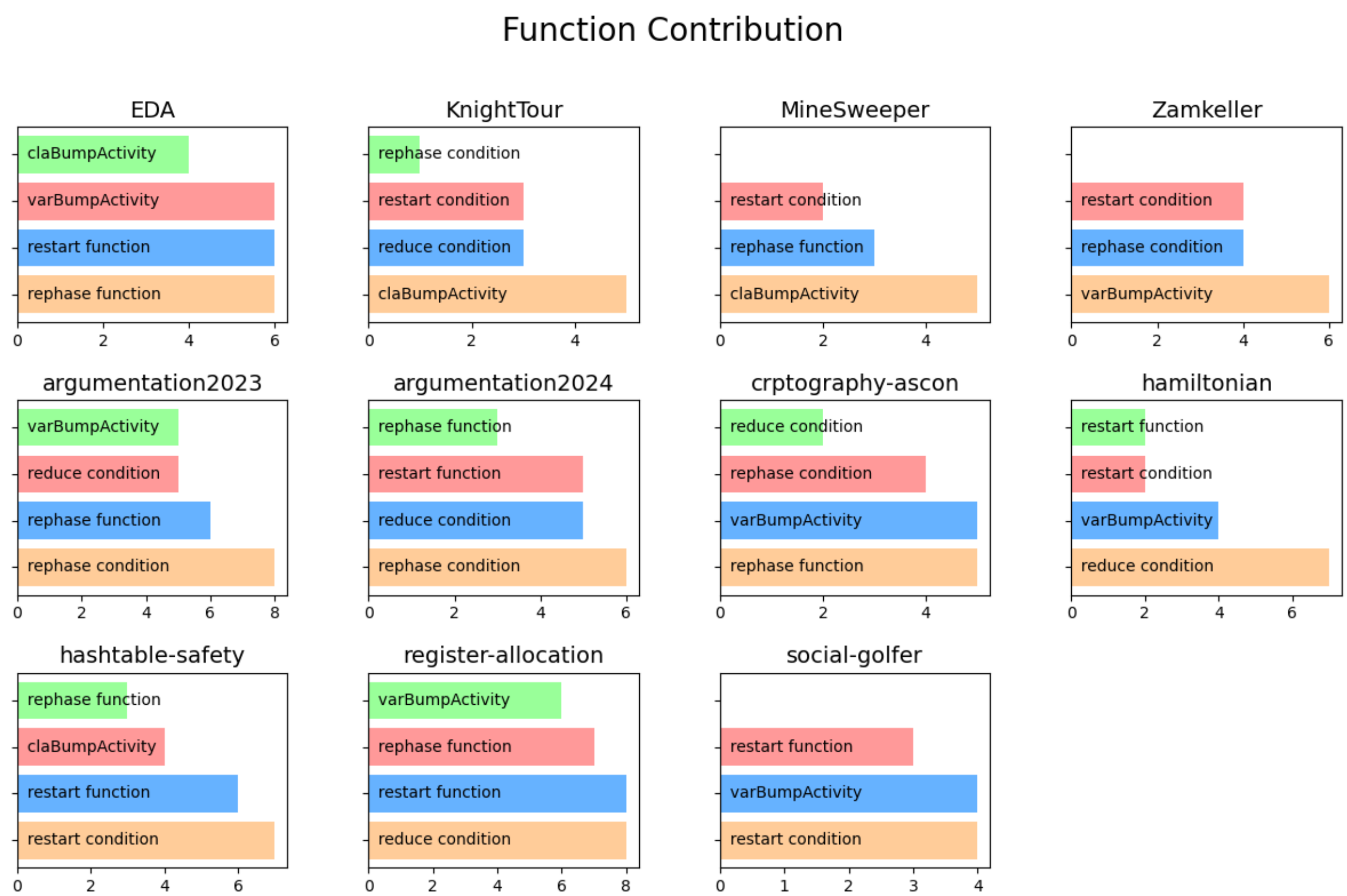

Supplementary Figure 6. Function contribution: where the vertical axis lists different function candidates and the horizontal axis denotes the frequency of contributions.

Notes: A $k \times k$ chessboard where a knight's tour is attempted, covering all squares and returning to the start point (a solution is not possible for odd-sized boards, i.e., they are unsatisfiable).

- **Zamkeller**:
  Parameter Θ: $\{k, n\}$;
  Parameter Space: $[3, 34] \times [25, 100]$;
  Notes: k represents the total sequence length, and n represents the subsequence length; For all subsequences of length k, the goal is to change them into the minimum number of distinct sequences.

The detailed configuration of the training dataset and the function candidates in heuristics discovery are presented in Supplementary Table 1.

4.2 Evaluation Metric

We consider two specific metrics for evaluating the performance of a SAT solver: (1) the number of SAT instances solved within the given timeout bound, and (2) the Penalized Average Runtime with a factor of 2 score (PAR-2). Both metrics are commonly used in the SAT Competitions.

Consider a dataset of n instances. Let t_i be the runtime of the SAT solver on the instance i. The PAR-2 score is formally defined as:

$$\text{PAR-2} = \frac{1}{n}\sum_{i=1}^{n} \tau_i \quad \text{where} \quad \tau_i = \begin{cases} t_i, & \text{if } t_i \leq \mathscr{T} \;\; (\mathscr{T} \text{ is the predefined timeout bound}) \\ 2\mathscr{T}, & \text{if } t_i > \mathscr{T} \text{ or the solver fails to return a result.} \end{cases}$$

For example, consider a benchmark dataset with three instances and a timeout bound $\mathscr{T} = 100$ seconds. The runtimes (in seconds) for the three instances are: $t_1 = 80$ for instance 1, $t_2 = 120$ for instance 2, the solver fails to return a result for instance 3. Then

Original Prompt

(Role) You are a SAT solver researcher trying to rewrite the {{ func_name }} function(s).

(Goal) Your goal is to improve the SAT solver by rewriting the {{ func_name }} function(s), after reading and understanding the <key code> of SAT solver below.

(Tips) Tips:
1) Your rewritten function code must start with ”’// start function name”’ and end with ”’// end function name”’
2) Your rewritten function(s) code must be different from original code, not just rewrite code synonymous!
3) You are not allowed to create your own new function(s) in the rewritten function(s). You are not allowed to create your own new global variables, but you can use the global variables existing in the <key code>.
4) Make sure the rewritten function(s) code can be executed correctly.

<key code> of SAT solver is:
{{ replace_key_code }}

Updated Prompt

(Role) You are a SAT solver researcher trying to improve the {{ func_name }} function.

(Goal) Objective:
Your goal is to improve the SAT solver by rewriting the {{ func_name }} function.

Instructions:
1. Carefully read and comprehend the <key code> of the SAT solver provided below.
2. Analyze potential improvements and devise a strategy for optimizing the heuristics of function.
3. Deliver your improved function(s) with the following format:
- Begin with: ‘// start function name‘
- End with: ‘// end function name‘

(Tips) Tips:
1. Ensure that your rewritten function(s) are substantially different from the original, beyond mere synonym replacements.
2. You may utilize existing global variables from the <key code>, but refrain from introducing new global variables.
3. Verify that the rewritten function(s) execute correctly.

Take a deep breath and think it step by step.

<key code> of SAT solver is:
""" {{ replace_key_code }} """ ...

Supplementary Figure 7. Comparison of original prompt and optimized prompt

- since $t_1 \leq \mathscr{T}$, we have $\tau_1 = 80$;
- since $t_2 > \mathscr{T}$, we have $\tau_2 = 200$ (penalized);
- since the solver fails for instance 3, we have $\tau_3 = 200$ (penalized).

Therefore, the PAR-2 score is given by

$$\text{PAR-2} = \frac{1}{3}(80+200+200) = \frac{480}{3} = 160.$$

Supplementary Table 1. Configuration of training set, where the indices 1 to 7 represent the following function candidates in order: rephase_condition, rephase_function, reduce_condition, restart_condition, restart_function, varBumpActivity, claBumpActivity.

Dataset	Training timeout (seconds)	Function candidate
cryptography-ascon	800	1, 2, 3, 6
register-allocation	2000	2, 3, 5, 6
social-golfer	2000	1, 4, 5, 6
hashtable-safety	500	2, 4, 5, 7
argumentation 2023	2000	1, 2, 3, 6
argumentation 2024	2000	1, 2, 3, 5
hamiltonian	800	3, 4, 5, 6
MineSweeper	500	2, 3, 4, 7
KnightTour	2000	1, 3, 4, 7
Zamkeller	2000	1, 3, 4, 6
EDA	800	2, 5, 6, 7

4.3 Search Space for Parameter Tuning

In this paper, we adopt SMAC3 to optimize the parameters of the SAT solvers across different datasets. The search space for each solver, including the parameter name, type, description and range, are presented in Supplementary Tables 2, 3, and 4.

Supplementary Table 2. ModSAT configuration parameters.

Parameter	Type	Description	Search Space
`var-decay`	double	Variable activity decay factor	$(0,1)$
`cla-decay`	double	Clause activity decay factor	$(0,1)$
`rnd-freq`	double	Frequency for random variable selection	$[0,1]$
`rnd-init`	bool	Randomize initial activities	$\{\text{true}, \text{false}\}$
`rfirst`	int	Base restart interval	$[1, 1e4]$
`rinc`	double	Restart interval increase factor	$(1.5, 4)$
`gc-frac`	double	Wasted memory fraction triggering garbage collection	$(0,1)$
`min-learnts`	int	Minimum learnt clause limit	$[0, 1e6]$

4.4 Comparison of Different LLMs

This subsection provides a detailed comparison of different LLMs. Four representative LLMs are tested, including: DeepSeek-V3, GPT-4o, Qwen2.5-Instruct, and Llama 3.3 70B-Instruct.

Firstly, in Supplementary Table 5 we compare their economic cost, parameters and context length. DeepSeek-V3 and GPT-4o are accessed through their official APIs, while Qwen 2.5-72B Instruct and Llama-3.3 are invoked via third-party APIs from SiliconFlow (https://siliconflow.cn/). From the pricing comparison, it is evident that open-source models offer a substantially lower cost per million tokens than proprietary, closed-source models.

Specifically, GPT-4o exhibits the highest price, with input and output costs of 2.50 and 10.00 per million tokens, respectively. DeepSeek-V3 is relatively more affordable, at 0.27 for input and 1.10 for output. Other open-source models such as Qwen 2.5-72B and Llama-3.3 are much cheaper than GPT-4o. Llama-3.3, in particular, achieves one of the lowest token costs (0.49 input, 0.80 output).

We compare the performance of different LLMs on the EDA dataset in Supplementary Table 6. As discussed in the Method section, AutoModSAT uses a *three-agent* loop: LLM coder modifies solver code; LLM evaluator identifies synonymous code; LLM repairer fixes bugs. Due to that the LLMs evaluator and repairer focus on correctness (not diversity), we fix their temperature to 0.2 in our framework. As for the LLMs coder, multiple temperatures $t \in \{0.2, 0.5, 0.7, 1.0\}$ are tested. Although the theoretical temperature range is $[0,2]$, in practice it is observed that when $t > 1.0$, the probability of generating correct code drops significantly. Therefore, we limit the upper bound of coder temperature to 1.0.

It can be seen that as the coder temperature increases, the fail rate of the generated code consistently grows, indicating that higher exploration makes the modifications more unstable. In contrast, PAR-2 improves when moving from 0.2 to intermediate temperatures and then degrades at 1.0. For DeepSeek-V3 and GPT-4o, the best PAR-2 is achieved at temperature 0.7, while

Supplementary Table 3. Kissat configuration parameters.

Parameter	Type	Description	Search Space
`chrono`	bool	Enable chronological backtracking	$\{0,1\}$
`eliminate`	bool	Enable variable elimination	$\{0,1\}$
`forcephase`	bool	Force initial phase assignment	$\{0,1\}$
`minimize`	bool	Enable clause minimization	$\{0,1\}$
`phase`	bool	Set initial decision phase	$\{0,1\}$
`phasesaving`	bool	Enable phase saving during restarts	$\{0,1\}$
`probe`	bool	Enable failed literal probing	$\{0,1\}$
`reduceint`	int	Conflict interval for clause DB reduction	$\{10^1,10^2,10^3,10^4,10^5\}$
`rephaseint`	int	Conflict interval for phase resetting	$\{10^1,10^2,10^3,10^4,10^5\}$
`restartint`	int	Base restart interval (conflicts)	$\{1,10^2,10^3,10^4\}$
`restartmargin`	int	Rapid restart margin threshold	$\{0,5,10,15,20,25\}$
`simplify`	bool	Enable periodic simplification	$\{0,1\}$
`stable`	int	Search stability mode (0: focused, 1: stable, 2: switching)	$\{0,1,2\}$
`target`	int	Target phase selection strategy (0: negative, 1: positive, 2: best)	$\{0,1,2\}$
`tier1`	int	Tier 1 glue limit for learned clauses	$\{2,3,4,5\}$
`tier2`	int	Tier 2 glue limit for learned clauses	$\{6,7,8,9,10,20,50\}$

Supplementary Table 4. CaDiCaL configuration parameters.

Parameter	Type	Description	Search Space
`chrono`	int	Chronological backtracking mode (0: none, 1: limited, 2: always)	$\{0,1,2\}$
`elim`	bool	Enables variable elimination during simplification	$\{0,1\}$
`forcephase`	bool	Forces phase saving for decision variables	$\{0,1\}$
`minimize`	bool	Enables clause minimization during conflict analysis	$\{0,1\}$
`phase`	bool	Initial decision phase assignment (0: negative, 1: positive)	$\{0,1\}$
`probe`	bool	Enables probing (failed literal detection)	$\{0,1\}$
`reduceint`	int	Conflict interval for clause database reduction	$\{10^2,10^3,10^4,10^5\}$
`rephaseint`	int	Conflict interval for resetting variable phases	$\{10^1,10^2,10^3,10^4,10^5\}$
`restartint`	int	Base restart interval (conflicts between restarts)	$\{2,10^2,10^3,10^4\}$
`restartmargin`	int	Restart margin percentage (Luby sequence scaling)	$\{0,5,10,15,20,25\}$
`stabilize`	bool	Stabilizes search by limiting activity updates	$\{0,1\}$
`target`	int	Search target (0: SAT, 1: UNSAT, 2: balanced)	$\{0,1,2\}$

Qwen-2.5 and Llama-3.3 obtain their minimum PAR-2 at 0.5. Overall, the coder performance of GPT-4o and DeepSeek-V3 is comparable and clearly stronger than that of Qwen-2.5 and Llama-3.3. The trends for the downstream evaluator and repairer agents are similar: GPT-4o attains the highest checking and repair correctness, followed by DeepSeek-V3, with Llama-3.3 and Qwen-2.5 forming a second tier. Considering both the performance and economic cost, we therefore adopt DeepSeek-V3 with coder temperature 0.7 as the default configuration in our main experiments. Note that we do not customize system prompts for all of these models. After fixing the model and temperature parameters, an experiment with 50 iterations consumes approximately 10 million tokens.

4.5 Ablation Study

Here we perform an ablation study to quantify the contribution of each component in AutoModSAT. We sequentially remove the three components: modularized SAT solver, presearch strategy, and automatic prompt optimization, to investigate their

Supplementary Table 5. Context length, parameter size, and pricing (per million tokens) for selected models.

Model	Context length	Parameter size	Official cost (/M tokens)
DeepSeek-V3	128k	671B	Input: $0.27; Output: $1.10
GPT-4o	128k	(not disclosed)	Input: $2.50; Output: $10.00
Qwen2.5-72B	128k	72.7B	Input: $1.40; Output: $5.60
Llama-3.3	128k	70B	Input: $0.49; Output: $0.80

Supplementary Table 6. Performance vs. coder temperature with correctness and time breakdown on EDA. AutoModSAT uses a three-agent loop (coder/evaluator/repairer). We fix evaluator/repairer temperature to 0.2 and sweep coder temperature $t \in \{0.2, 0.5, 0.7, 1.0\}$. We report PAR-2, evaluator checkness, failure rate, repair correctness, and single call time of the agent.

Model	Coder Temperature	Coder time (s)	PAR-2	Evaluator checkness (%)	Evaluator time (s)	Fail rate (%)	Repair correctness (%)	Repair time (s)
DeepSeek-V3	0.2	21.05 ± 0.55	508.73	36.10	20.00 ± 0.91	7.85	68.42	20.37 ± 0.51
DeepSeek-V3	0.5		389.56	12.34		10.92	77.03	
DeepSeek-V3	0.7		379.01	19.12		22.84	76.41	
DeepSeek-V3	1.0		646.18	6.27		41.63	82.55	
GPT-4o	0.2	25.08 ± 1.62	411.22	55.44	22.07 ± 2.90	2.31	87.26	21.41 ± 1.54
GPT-4o	0.5		394.08	31.67		11.28	85.91	
GPT-4o	0.7		378.67	28.54		13.27	85.12	
GPT-4o	1.0		449.79	9.83		32.44	81.73	
Qwen-2.5	0.2	20.61 ± 0.58	533.95	44.73	18.67 ± 0.89	2.12	64.88	20.12 ± 0.74
Qwen-2.5	0.5		514.37	30.12		12.83	73.76	
Qwen-2.5	0.7		521.74	16.41		25.09	82.34	
Qwen-2.5	1.0		672.66	7.55		44.38	79.68	
Llama-3.3	0.2	20.55 ± 0.58	642.18	53.88	19.64 ± 0.88	5.67	53.92	19.09 ± 0.55
Llama-3.3	0.5		421.03	29.45		13.77	81.15	
Llama-3.3	0.7		436.01	15.72		16.21	70.73	
Llama-3.3	1.0		685.54	2.83		35.91	68.54	

respective contributions to the performance. The results are summarized in Supplementary Table 7.

- **AutoModSAT without Modularization.** Here we apply the AutoModSAT pipeline to the original, non-modularized SAT solver (i.e., MiniSat with rephasing heuristics). The most significant degradation in PAR-2 is observed among all ablations. Without modularization, LLMs struggle to generate correct and diverse heuristics, which limits the solver improvement. These results show that modularization is not merely an auxiliary enhancement but a foundational requirement for enabling effective LLM-driven solver optimization.
- **AutoModSAT without presearch strategy.** In this variant, the LLM-based agents operate directly on the full set of function candidates without the presearch strategy. It can be seen that searching over the full space indeed leads to a slight increase in PAR-2.
- **AutoModSAT without prompt optimization.** Here we replace the optimized coder prompts with manually written baseline prompts. Removing automatic prompt optimization consistently reduces the solver performance. This supports the common sense that high-quality proper prompts are very important for LLMs to work desirably.

Overall, the modularized SAT solver offers the largest performance gains by enabling reliable LLM-driven code generation, while presearch strategy and prompt optimization further enhance the stability and efficiency.

In Supplementary Figure 8, we also report the convergence behavior of AutoModSAT with and without presearch. It clearly shows that after 50 iteration, AutoModSAT with presearch can achieve a substantially lower PAR-2 score than that without presearch. This also means that presearch can improve the computational efficiency of AutoModSAT.

4.6 Generalization Tests

In-domain generalization tests. To further distinguish domain specialization from structural overfitting, we conduct the generalization tests where the training and test data share the same domain. The new test data are generated as follows. First,

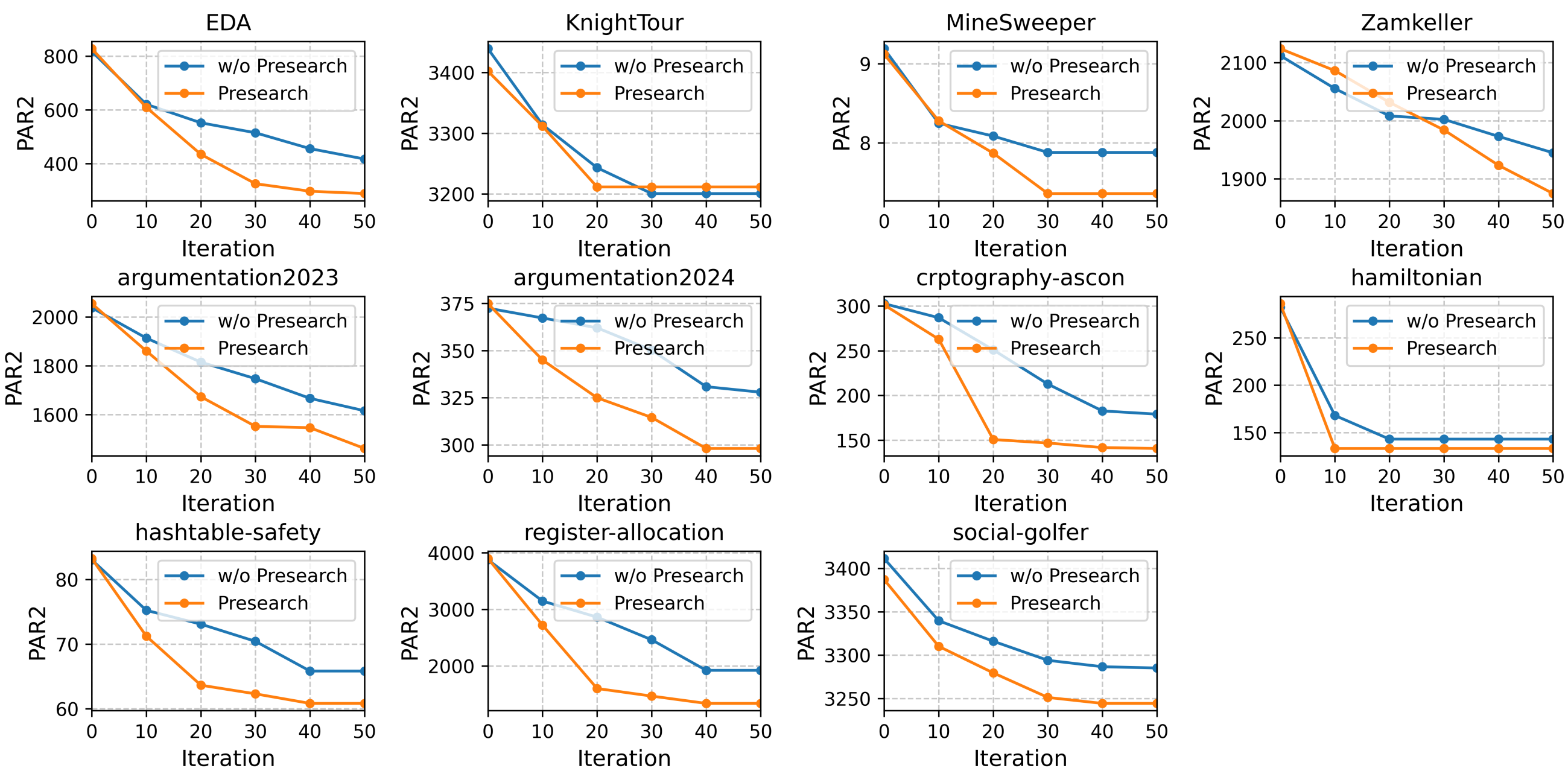

Supplementary Figure 8. Convergence behavior of AutoModSAT with and without presearch.

among the seven families used in the solver optimization, three families have appeared repeatedly in the SAT Competitions and thus contain additional instances beyond those from the SAT Competitions 2023 and 2024; the other four families do not have additional instances. These three families are argumentation (156 instances), hamiltonian (1060 instances), and social-golfer (19 instances). For argumentation and hamiltonian, we randomly sample 100 previously unseen instances as the test data, while for social-golfer we use all 19 instances. Second, for the two families generated by Picat (MineSweeper, KnightTour), we sample extra 88 and 56 instances per family as the test data.

The generalization test results are shown in Supplementary Table 8. It can be observed that the heuristics discovered on argumentation, hamiltonian and social-golfer remain consistently competitive on their in-domain test data, and the same trend can be observed for the Picat-generated families. This indicates that as an automation framework of domain-specific solvers, the optimization by AutoModSAT does not merely memorize the training data, but can generalize well on the test data in the same domain.

Cross-domain generalization tests. Further generalization tests on the cross-domain data have been conducted and results are presented in Supplementary Table 9 and Supplementary Table 10. Overall, there are very few heuristics which can generalize cross the domain, which is natural given that the datasets differ from each other substantially and the optimization is conducted on particular dataset. Nonetheless, certain heuristics exhibit broader applicability: the heuristic discovered in hamiltonian achieves consistently competitive performance across multiple other datasets, indicating that some discovered strategies encode reusable search behaviors beyond their source domain.

4.7 Correctness Verification

After discovering heuristics in a SAT solver, we explicitly validate the correctness of the generated solvers over all the problem instances.

- **SAT case validation.** For every instance reported as satisfiable, the solver outputs a full assignment to all relevant variables. We then independently validate this assignment by substituting it back into the original CNF formula to see whether every clause is satisfied. This checker is implemented as a separate, simple CNF evaluator, and any instance for which the reported assignment does not satisfy the CNF is flagged as incorrect and the corresponding solver variant is rejected by AutoModSAT.
- **UNSAT proof verification.** For every instance reported as unsatisfiable, the solver is required to emit a DRAT proof. We verify this proof using the external proof checker drat-trim[13], which independently validates that the DRAT sequence constitutes a correct refutation of the original CNF. If drat-trim rejects the proof, the UNSAT result is deemed invalid and the corresponding solver mutation is rejected by AutoModSAT.

Supplementary Table 7. Ablation Study. Newly generated PAR-2 scores (lower is better) and solved counts in parentheses (higher is better) across 11 datasets for an ablation study. Bold values indicate the best PAR-2 score.

Dataset	ModSAT	AutoModSAT w/o modularization	AutoModSAT w/o presearch	AutoModSAT w/o prompt optimization	AutoModSAT
cryptography-ascon	208.60 (20)	200.45 (20)	178.30 (20)	180.12 (20)	**140.72 (20)**
register-allocation	7213.23 (6)	5120.10 (10)	1920.55 (17)	3055.32 (14)	**1177.80 (18)**
social-golfer	7836.30 (5)	7602.11 (5)	7420.24 (6)	7588.92 (5)	**7265.45 (6)**
hashtable-safety	83.25 (20)	80.14 (20)	67.92 (20)	78.50 (20)	**60.82 (20)**
argumentation 2023	4695.25 (13)	4002.33 (13)	3310.25 (14)	3535.10 (14)	**3229.65 (14)**
argumentation 2024	2745.57 (17)	780.22 (20)	305.70 (21)	465.41 (21)	**232.14 (21)**
hamiltonian	577.48 (38)	355.20 (39)	162.90 (40)	310.48 (39)	**133.13 (40)**
MineSweeper	9.12 (88)	9.06 (88)	7.88 (88)	9.12 (88)	**7.36 (88)**
KnightTour	8769.77 (7)	8620.22 (8)	8088.31 (11)	8120.50 (11)	**7815.68 (13)**
Zamkeller	3485.35 (54)	2622.10 (63)	2490.44 (62)	2455.80 (64)	**2053.21 (66)**
EDA	825.36 (46)	825.36 (46)	510.12 (48)	722.44 (46)	**376.01 (49)**

Supplementary Table 8. In-domain generalization tests. This table shows PAR-2 (solved instances in parentheses) of the produced solvers tested on the new in-domain data. Note that the argumentation dataset uses solvers optimized on argumentation 2023. Bold values indicate the best PAR-2 score.

Dataset	MiniSat	ModSAT	ModSAT para	Kissat	Kissat para	CaDiCaL	CaDiCaL para	AutoModSAT
social-golfer	5100.23 (10)	4805.77 (11)	4552.61 (12)	4321.08 (13)	4288.90 (13)	4269.09 (13)	4154.32 (13)	**4067.68 (14)**
argumentation	8750.66 (24)	6420.14 (41)	6012.45 (44)	5755.32 (45)	5488.76 (48)	5597.02 (47)	5324.11 (50)	**5083.01 (54)**
hamiltonian	980.44 (91)	820.15 (92)	690.73 (94)	640.20 (95)	410.55 (97)	560.34 (96)	245.67 (98)	**118.39 (100)**
MineSweeper	10.42 (88)	9.31 (88)	8.07 (88)	148.76 (88)	49.28 (88)	56.14 (88)	44.02 (88)	**7.51 (88)**
KnightTour	9198.35 (5)	8794.12 (7)	8127.46 (11)	8771.08 (7)	8599.41 (8)	8386.27 (10)	8310.58 (10)	**7864.93 (13)**

- **Cross solver consistency.** Finally, for each dataset, we compare the SAT/UNSAT status reported by all tested solvers (baseline solvers and AutoModSAT-discovered solvers). In our experiments, no inconsistencies in SAT/UNSAT status were observed across the solvers, and all reported SAT assignments and UNSAT proofs successfully passed the above validation pipeline.

5 Analysis of Discovered Heuristics

In this section, to demonstrate LLMs' ability of generating effective heuristics, we provide more contrastive examples (one for each function candidate), along with the explanations for the changes.

5.1 Examples of Discovered Heuristics

Supplementary Figure 9 provides an example for the updated claBumpActivity function, which introduces two key enhancements in contrast to the original implementation. First, during activity rescaling, it enforces a minimum activity threshold (min_activity = 1e-20) to prevent clauses from becoming numerically insignificant after scaling. This preserves the relevance of historically important clauses and avoids premature elimination from the learning process. Second, it incorporates dynamic decay adjustment based on recent conflict rates: when conflicts exceed 1000 and the LBD queue surpasses 50 entries, cla_inc is scaled down proportionally to the conflict density (with a floor of 0.8). This procedure adaptively moderates the activity growth during high-conflict phases, prioritizing recent high-impact clauses while maintaining stability. Together, these refinements yield a more balanced clause management strategy which utilizes valuable learned clauses while dynamically optimizes activity decay for solver efficiency.

Supplementary Figure 10 provides an example of the updated varBumpActivity function, which introduces three key improvements over the original. First, it scales the increment by (1.0 + 0.1 * decisionLevel()), prioritizing variables involved in recent decisions to accelerate conflict-driven learning. Second, the rescaling mechanism uses a larger threshold (1e100) and finer scale factor (1e-100), while preserving variable relevance by enforcing a minimum activity floor (1e-100) to maintain relative ordering and prevent premature underflow. Third, it optimizes heap management through conditional updates: dynamically adjusting the variable's heap position only when its activity exceeds the current maximum, or inserting undefined decision variables lazily. These enhancements collectively improve search guidance, reduce floating-point stability issues, and minimize unnecessary data structure operations.

Supplementary Table 9. Cross-domain generalization tests (part 1). This table shows PAR-2 of the solver discovered from one dataset but evaluated on the other datasets (row: source dataset, column: evaluation dataset).

Dataset	cryptography-ascon	register-allocation	social-golfer	hashtable-safety	argumentation 2023
cryptography-ascon	140.72 (20)	10000.00 (0)	9078.14 (2)	566.76 (20)	7618.30 (6)
register-allocation	591.36 (20)	1177.80 (18)	10000.00 (0)	175.23 (20)	3734.85 (14)
social-golfer	2770.50 (15)	9646.80 (1)	7265.45 (6)	100.85 (20)	4199.48 (13)
hashtable-safety	442.36 (20)	9621.51 (1)	10000.00 (0)	60.82 (20)	3472.21 (14)
argumentation 2023	238.68 (20)	8655.26 (3)	10000.00 (0)	135.31 (20)	3229.65 (14)
argumentation 2024	2857.93 (15)	8627.86 (3)	10000.00 (0)	6118.72 (10)	3273.99 (14)
hamiltonian	693.21 (20)	9040.47 (2)	7160.60 (6)	567.41 (20)	3434.80 (14)
MineSweeper	352.43 (20)	8674.13 (3)	10000.00 (0)	196.22 (20)	4181.09 (13)
KnightTour	691.36 (20)	8823.31 (3)	10000.00 (0)	215.33 (20)	3483.80 (14)
Zamkeller	339.15 (20)	9197.00 (2)	10000.00 (0)	67.09 (20)	8585.55 (4)
EDA	694.26 (20)	9602.40 (1)	10000.00 (0)	233.31 (20)	4281.80 (13)

Supplementary Table 10. Cross-domain generalization tests (part 2). This table shows PAR-2 of the solver discovered from one dataset but evaluated on the other datasets (row: source dataset, column: evaluation dataset).

Dataset	argumentation 2024	hamiltonian	MineSweeper	KnightTour	Zamkeller	EDA
cryptography-ascon	5451.93 (12)	2406.25 (35)	7.59 (88)	8624.27 (8)	7597.07 (22)	749.19 (47)
register-allocation	873.64 (21)	657.01 (39)	8.33 (88)	8290.95 (10)	5792.07 (37)	809.75 (47)
social-golfer	1733.74 (19)	267.90 (40)	7.51 (88)	8396.78 (9)	6567.69 (30)	609.15 (48)
hashtable-safety	516.20 (21)	260.45 (40)	7.62 (88)	8588.75 (8)	7721.79 (23)	557.97 (48)
argumentation 2023	379.91 (21)	253.03 (40)	8.44 (88)	8588.50 (8)	8000.07 (21)	405.91 (48)
argumentation 2024	232.14 (21)	768.64 (39)	10.45 (88)	8098.60 (11)	7969.48 (18)	2269.94 (41)
hamiltonian	863.64 (21)	133.13 (40)	8.49 (88)	8280.72 (10)	4711.09 (47)	2757.74 (38)
MineSweeper	1073.23 (20)	257.87 (40)	7.36 (88)	8498.32 (9)	7594.35 (21)	839.55 (47)
KnightTour	637.32 (21)	227.11 (40)	8.22 (88)	7815.68 (13)	7592.04 (21)	1022.73 (47)
Zamkeller	7589.31 (6)	4219.22 (29)	8.14 (88)	8153.99 (11)	2053.21 (66)	388.61 (49)
EDA	988.32 (20)	239.86 (40)	8.30 (88)	8590.95 (8)	4397.11 (49)	376.01 (49)

Supplementary Figure 11 provides an example of the updated restart_condition function. It improves upon the original one by replacing the static threshold approach with a dynamic, performance-driven restart strategy that adapts to real-time solver behavior. Instead of relying on fixed queue sizes and hard coded multipliers, the new version calculates the restart thresholds using multiple runtime metrics: it combines average LBD (measuring clause quality) with conflict rates (tracking solver progress) to dynamically adjust the restart timing based on problem difficulty. Crucially, it introduces a progress-sensitive mechanism that aggressively lowers thresholds when stagnation is detected (progressEstimate changes < 0.01), enabling proactive recovery from plateaus, a capability absent in the original. This multi-factor approach yields more precise restart decisions, reduces wasteful computations, and enhances solver adaptability across diverse SAT instances while maintaining robustness during initialization through default thresholds.

Supplementary Figure 12 provides an example of the updated restart_function. Unlike the original version which always resets to decision level 0 (a full restart), the enhanced function dynamically calculates two exponential moving averages of conflict difficulty (fast_avg and slow_avg) using Literal Block Distance (LBD) scores. By analyzing the ratio between these averages, it intelligently selects one of three restart depths: full restart (level 0) for deteriorating conflict quality, partial restart (mid-level) for moderately harder conflicts, or minimal restart (current level -1) for stable conditions. This adaptability preserves useful learned clauses during partial/minimal restarts and reduces redundant recomputation. Additionally, periodic clause database reduction (every 16 restarts) curbs memory growth, while rebuilding the variable order heap ensures that the branching decisions can reflect the updated activity scores.

Supplementary Figure 13 provides an example of the updated rephase_condition function. In contrast to the prior version which solely relies on a fixed rephase limit, the new implementation dynamically adjusts rephasing intervals based on real-time search progress and conflict density. By calculating normalized progress through trail size changes and setting variable-driven thresholds (e.g., 2% of total variables), it detects stagnation when progress falls below expectation and then reduces the subsequent rephase intervals exponentially. On the other hand, substantial progress triggers gradual interval expansion. This self-tuning capability optimizes computational efficiency: it minimizes unnecessary rephasing during the search loop, thereby improving solution convergence.

Supplementary Figure 14 provides an example of the updated rephase_function. First, it implements dynamic rephase limit adjustment by scaling rephase_limit based on progress measured through conflict resolution (conflictR). If progress

occurs, the limit increases by 50% to exploit productive phases more aggressively; otherwise, it decays by 10% (with a lower bound of 512) to conserve resources during stagnation. This replaces the original static increment (+= 8192) and fixed decay (threshold *= 0.9), enabling context-sensitive resource allocation. Second, the refined phase selection strategy uses weighted probabilities with four distinct policies instead of the original rigid three-policy cascade: local-best phases (40%), global phase inversion (30%), randomized phases for low-activity variables (20%), and user-specified phases (10%). In addition, the introduction of randomization for less-active variables helps escape local optima. Finally, adaptive threshold dynamically scales with the solvers state (threshold = trail.size() * 0.8).

Supplementary Figure 15 provides an example of the updated reduce_condition function, which enhances the original version through four key improvements. First, it retains the core check for absolute learnt clause limits (learnts.size() >= max_learnts), ensuring baseline constraint adherence. Second, it introduces memory pressure awareness by triggering reduction when wasted clause memory exceeds 80% of the garbage collection threshold (ca.wasted() > ca.size() * garbage_frac * 0.8). This proactively mitigates memory bloat and improves cache efficiency. Third, a learnt-to-original clause ratio check (learnts.size() > 2 * nClauses()) prevents learnt clauses from disproportionately dominating the formula, maintaining balanced reasoning. Finally, a conflict-driven heuristic (conflictR > 1000 && learnts.size() > max_learnts * 0.8) adapts to high-conflict phases by initiating earlier reduction, thus accelerating recovery from solver stagnation. These criteria boost robustness: they minimize redundant computation through memory-sensitive garbage collection, preserve clause quality via ratio controls, and dynamically respond to runtime behavior.

5.2 Novelty Assessment of Discovered Heuristics

To address questions regarding the fundamental novelty of the heuristics discovered by AutoModSAT, we conduct a systematic investigation. It should be noted that an evaluation of the true novelty is challenging due to the infeasibility of exhaustively enumerating all open-source codes. Our goal is therefore to perform a rigorous comparison against a well-defined corpus of modern SAT solvers. The assessment process is comprised of three key steps:

- **Solver collection.** We collect the top-10 solvers from major track in SAT competitions between 2020 and 2025 to establish a representative benchmark of state-of-the-art techniques.
- **Heuristic extraction.** For each solver, we identify its source files with an LLM to parse the code, identify heuristic rules, and normalize their descriptions into a consistent format.
- **Automated comparison.** We adopt an LLM-as-a-judge strategy to automatically compare the heuristics discovered by AutoModSAT with those extracted from the reference corpus. The comparison follows a binary (True/False) format, evaluating whether a given heuristic shares similar ideas or mechanisms with any in the corpus. Heuristics marked as "similar ideas" are then subject to manual verification by human experts to confirm or refute conceptual overlap.

Finally, we have obtained 60 solver repositories and sorted out the seven heuristic categories from each solver into structured files. After running the automated comparison, we find out that some solvers that potentially share similar ideas with the discovered heuristics by AutoModSAT, which is shown in Supplementary Table 11. However, further manual verification confirms that none of these heuristics exhibit completely the same implementations.

Notably, compared with the original heuristics, the heuristics discovered by AutoModSAT tend to integrate multiple rules, resulting in more complex and hybrid heuristics. While in a few instances, it is likely that some rules draw inspiration from prior solvers. For example, the average LBD computation used in restart conditions appears in the Maple family of solvers. However, other key elements such as the incorporation of a `progressEstimate` factor do not appear in any existing implementations. Overall, these findings suggest that there are new heuristics discovered by AutoModSAT.

Supplementary Table 11. Solvers which share some similar ideas with the discovered heuristics.

Heuristics	Solvers with similar idea
claBumpActivity	Maple_MBDR_Cent_PERM
reduce_condition	MapleLCMDistChrBt-DL<br>kissat-sc2022-bulk<br>kissat_inc
rephase_condition	kissat-sc2024<br>BreakID-kissat<br>Kissat-CURE<br>kissat-sat_crvr_gt<br>Kissat_MAB_prop<br>Kissat_MAB-DC
rephase_function	kissat-sc2020-default<br>CaDiCal-PriPro<br>kissat-pred
restart_condition	Maple_simp21<br>Maple_MBDR_Cent_PERM<br>Maple_MBDR_BJL7_Loc<br>Maple_MBDR_BJL6_Tie
restart_function	kissat-sc2025<br>CaDiCal-PriPro<br>kissat-1.0.3<br>Maple_MBDR_BJL7_Loc<br>kissat-pred
varBumpActivity	Maple_MBDR_Cent_PERM<br>kissat-1.0.3<br>kissat-pred<br>CaDiCal

original claBumpActivity

```
inline void Solver::claBumpActivity (Clause& c) {
        if ( (c.activity() += cla_inc) > 1e20 ) {
            // Rescale:
            for (int i = 0; i < learnts.size(); i++)
                ca[learnts[i]].activity() *= 1e-20;
            cla_inc *= 1e-20; }
        }
```

discovered claBumpActivity

```
inline void Solver::claBumpActivity (Clause& c) {
    // Adaptive scaling based on clause length and conflict participation
    if ((c.activity() += cla_inc) > 1e20) {
        // Maintain minimum activity while scaling to preserve relevance
        double scale_factor = 1e-20;
        double min_activity = 1e-20;
        for (int i = 0; i < learnts.size(); i++) {
            ca[learnts[i]].activity() *= scale_factor;
            if (ca[learnts[i]].activity() < min_activity)
                ca[learnts[i]].activity() = min_activity;
        }
        cla_inc *= scale_factor;

        // Stabilize scaling by maintaining lower bound on clause activity
        if (cla_inc < min_activity)
            cla_inc = min_activity;
    }
    // Dynamic decay adjustment based on recent conflict rate
    if (conflicts > 1000 && lbd_queue_size > 50) {
        double conflict_scale = 1.0 - 0.01 * (lbd_queue_size / 50.0);
        cla_inc *= conflict_scale > 0.8 ? conflict_scale : 0.8;
    }
}
```

Supplementary Figure 9. Example of LLM discovered heuristics of claBumpActivity

original varBumpActivity

```
inline void Solver::varBumpActivity(Var v, double inc) {
    activity[v] += inc;
    // Rescale condition to prevent floating-point overflow earlier
    if (activity[v] > 1e50) {
        for (int i = 0; i < nVars(); i++)
            activity[i] *= 1e-50;
        var_inc *= 1e-50;
        }
    }
```

discoverd varBumpActivity

```
inline void Solver::varBumpActivity(Var v, double inc) {
    // Scale the increment based on decision level to prioritize recent variables
    double scaled_inc = inc * (1.0 + 0.1 * decisionLevel());
    activity[v] += scaled_inc;
    // More gradual rescaling that preserves relative ordering better
    if (activity[v] > 1e100) {
        double scale_factor = 1e-100;
        for (int i = 0; i < nVars(); i++) {
            activity[i] *= scale_factor;
            // Prevent variables from becoming completely inactive
            if (activity[i] < 1e-100) activity[i] = 1e-100;
        }
        var_inc *= scale_factor;
    }
    // Optimized heap update with early exit for common case
    if (order_heap.inHeap(v)) {
        if (activity[v] > activity[order_heap[0]]) {
            order_heap.decrease(v);
        }
    } else if (decision[v] && value(v) == l_Undef) {
        order_heap.insert(v);
    }
}
```

Supplementary Figure 10. Example of LLM discovered heuristics of varBumpActivity

original restart_condition

```
bool Solver::restart_condition(){
    if ((lbd_queue_size == 50 && 0.8 * fast_lbd_sum / lbd_queue_size > slow_lbd_sum /
    conflicts) || !withinBudget())
        return true;
    else
        return false;

}
```

discoverd restart_condition

```
bool Solver::restart_condition() {
    // Base restart condition on conflict count
    if (conflicts <= 0) return false;

    // Dynamic restart threshold based on recent solver performance
    double restart_threshold;

    if (lbd_queue_size > 0) {
        // Use both average LBD and conflict rate for adaptive restart
        double avg_lbd = fast_lbd_sum / lbd_queue_size;
        double conflict_rate = (double)conflictR / (double)conflicts;

        // Adjust threshold based on problem difficulty (higher LBD = harder problem)
        restart_threshold = restart_first * (0.8 + 0.4 * avg_lbd) * (1.0 + 0.5 *
    conflict_rate);

        // More aggressive restart if we're making little progress
        if (progressEstimate() - last_rephase_progress < 0.01) {
            restart_threshold *= 0.7;
        }
    } else {
        // Default threshold for initial phase
        restart_threshold = restart_first;
    }

    // Check if we've reached the threshold
    if (conflictR >= restart_threshold) {
        conflictR = 0;
        return true;
    }
    return false;
}
```

Supplementary Figure 11. Example of LLM discovered heuristics of restart condition

original restart_function

```
void Solver::restart_function(){
    fast_lbd_sum = lbd_queue_size = lbd_queue_pos = 0;
    int level = 0;
    cancelUntil(level);
}
```

discovered restart_function

```
void Solver::restart_function() {

    // Update restart statistics
    static double fast_avg = 0, slow_avg = 0;
    static int restart_count = 0;

    // Calculate moving averages of LBD scores
    if (lbd_queue_size > 0) {
        double curr_fast = fast_lbd_sum / lbd_queue_size;
        fast_avg = 0.9 * fast_avg + 0.1 * curr_fast;
        slow_avg = 0.99 * slow_avg + 0.01 * curr_fast;
    }

    // Determine restart level based on recent performance
    int restart_level = 0;
    if (fast_avg > 0 && slow_avg > 0) {
        double ratio = fast_avg / slow_avg;
        if (ratio > 1.2) {
            // Recent conflicts are harder - do a full restart
            restart_level = 0;
        } else if (ratio > 1.0) {
            // Partial restart - keep some learned clauses
            restart_level = std::max(0, decisionLevel() / 2);
        } else {
            // Recent conflicts are easier - minimal restart
            restart_level = std::max(0, decisionLevel() - 1);
        }
    }

    // Reset LBD statistics
    fast_lbd_sum = lbd_queue_size = lbd_queue_pos = 0;

    // Perform the restart
    cancelUntil(restart_level);

    // Occasionally reduce the clause database after restarts
    if (restart_count++ % 16 == 15) {
        reduceDB();
    }

    // Rebuild the order heap to reflect any activity changes
    rebuildOrderHeap();
}
```

Supplementary Figure 12. Example of LLM discovered heuristics of restart function

original rephase_condition

```
bool Solver::rephase_condition() {
    if (rephases >= rephase_limit) return true;
    else return false;
}
```

discovered rephase_condition

```
bool Solver::rephase_condition() {
    // Adaptive rephasing based on progress and conflict density
    const int base_rephase_limit = 1024;
    const double progress_factor = 0.02;  // 2% of total variables

    if (conflictR < rephase_limit)
        return false;

    // Calculate normalized progress since last rephase
    int progress = trail.size() - last_rephase_progress;
    int progress_threshold = std::max(50, (int)(nVars() * progress_factor));

    // Dynamic limit adjustment based on search progress
    if (progress < progress_threshold) {
        // Stagnation detected - reduce next rephase interval
        rephase_limit = std::max(base_rephase_limit, rephase_limit * 2/3);
    } else {
        // Good progress - expand next rephase interval
        rephase_limit = std::min(base_rephase_limit * 16, rephase_limit * 3/2);
    }

    // Reset conflict counter and update progress tracker in rephase_function
    return true;
}
```

Supplementary Figure 13. Example of LLM discovered heuristics of rephase condition

original rephase_function

```
void Solver::rephase_function() {
    int var_nums = nVars();
    conflictR = 0, rephases = 0, threshold *= 0.9, rephase_limit += 8192;

    int phase_rand = rand() % 100;
    if ((phase_rand -= 40) < 0){
        for (int i = 0; i < var_nums; i++){
            polarity[i] = local_best[i];
        }
    }
    else if ((phase_rand -= 25) < 0){
        for (int i = 0; i < var_nums; i++){
            polarity[i] = 1-local_best[i];
        }
    }
    else if ((phase_rand -= 15) < 0){
        for (int i = 0; i < var_nums; i++){
            polarity[i] = !polarity[i];
        }
    }
    else {
        for (int i = 0; i < var_nums; i++){
            polarity[i] = saved[i];
        }
    }
}
```

discovered rephase_function

```
void Solver::rephase_function() {
    // Adaptive rephase limit adjustment
    if (rephases > 0 && conflictR > last_rephase_progress) {
        // Progress was made - be more aggressive with rephasing
        rephase_limit = rephase_limit * 1.5;
    } else {
        // No progress - be more conservative
        rephase_limit = rephase_limit * 0.9;
        if (rephase_limit < 512) rephase_limit = 512;
    }
    last_rephase_progress = conflictR;
    rephase_count++;

    // Dynamic phase selection strategy
    double rand_val = drand(random_seed);
    if (rand_val < 0.4) {
        // 40% chance: Use local best phases
        for (int v = 0; v < nVars(); v++) {
            polarity[v] = local_best[v];
        }
        if (verbosity >= 1) printf("| Rephase: Local best phases |\n");
    } else if (rand_val < 0.7) {
        // 30% chance: Invert all phases
        for (int v = 0; v < nVars(); v++) {
            polarity[v] = !polarity[v];
        }
        if (verbosity >= 1) printf("| Rephase: Inverted all phases |\n");
    } else if (rand_val < 0.9) {
        // 20% chance: Random phases for variables with low activity
        double activity_threshold = 0.2 * var_inc;
        for (int v = 0; v < nVars(); v++) {
            if (activity[v] < activity_threshold) {
                polarity[v] = drand(random_seed) < 0.5;
            }
        }
        if (verbosity >= 1) printf("| Rephase: Randomized low-activity phases |\n");
    } else {
        // 10% chance: Original user phases
        for (int v = 0; v < nVars(); v++) {
            if (user_pol[v] != l_Undef) {
                polarity[v] = (user_pol[v] == l_True);
            }
        }
        if (verbosity >= 1) printf("| Rephase: Reset to user phases |\n");
    }

    // Reset the threshold for next local best phase tracking
    threshold = trail.size() * 0.8;
    cancelUntil(0);
}
```

Supplementary Figure 14. Example of LLM discovered heuristics of rephase function

original reduce_condition

```
bool Solver::reduce_condition()
{
    if (learnts.size() >= max_learnts)  return true;
    else return false;
}
```

discovered reduce_condition

```
bool Solver::reduce_condition()
{
    // Check if we've reached the absolute limit of learnt clauses
    if (learnts.size() >= max_learnts) return true;

    // Consider memory pressure and garbage collection needs
    if (ca.wasted() > ca.size() * garbage_frac * 0.8) return true;

    // Consider the ratio between learnt and original clauses
    if (learnts.size() > 0 && learnts.size() > 2 * nClauses()) return true;

    // Consider recent solver performance (conflict rate)
    if (conflictR > 1000 && learnts.size() > max_learnts * 0.8) return true;

    return false;
}
```

Supplementary Figure 15. Example of LLM discovered heuristics of reduce condition

Supplementary References

1. Marques-Silva, J. P. & Sakallah, K. A. Grasp: A search algorithm for propositional satisfiability. *IEEE Transactions on Comput.* **48**, 506–521 (1999).
2. Biere, A., Heule, M., van Maaren, H. & Walsh, T. Conflict-driven clause learning sat solvers. *Handb. Satisf. Front. Artif. Intell. Appl.* **4**, 131–153 (2009).
3. Liang, J. H., Ganesh, V., Poupart, P. & Czarnecki, K. Learning rate based branching heuristic for SAT solvers. In *Theory and Applications of Satisfiability Testing–SAT 2016: 19th International Conference*, 123–140 (2016).
4. Ramos, A., Van Der Tak, P. & Heule, M. J. Between restarts and backjumps. In *Theory and Applications of Satisfiability Testing-SAT 2011: 14th International Conference, SAT*, 216–229 (2011).
5. Luby, M., Sinclair, A. & Zuckerman, D. Optimal speedup of las vegas algorithms. *Inf. Process. Lett.* **47**, 173–180 (1993).
6. Audemard, G. & Simon, L. Glucose 2.1: Aggressive, but reactive, clause database management, dynamic restarts. In *Pragmatics of SAT 2012* (2012).
7. Biere, A. & Fröhlich, A. Evaluating cdcl restart schemes. In *Proceedings of Pragmatics of SAT*, 1–17 (2015).
8. Biere, A. Lingeling, plingeling, picosat and precosat at sat race 2010. *FMVReport Ser. Tech. Rep.* **10** (2010).
9. Jeroslow, R. G. & Wang, J. Solving propositional satisfiability problems. *Annals mathematics Artif. Intell.* **1**, 167–187 (1990).
10. Soos, M. Strangenight. In *Proceedings of SAT Competition 2013: Solver, Benchmark and Proof Checker Descriptions*, 89–90 (Department of Computer Science, University of Helsinki, 2013).
11. Balint, A., Belov, A., Järvisalo, M. & Sinz, C. Overview and analysis of the sat challenge 2012 solver competition. *Artif. Intell.* **223**, 120–155 (2015).
12. Zhou, N.-F., Kjellerstrand, H. & Fruhman, J. *Constraint solving and planning with Picat*, vol. 11 (Springer, 2015).
13. Wetzler, N., Heule, M. J. H. & Jr., W. A. H. Drat-trim: Efficient checking and trimming using expressive clausal proofs. In *Theory and Applications of Satisfiability Testing*, vol. 8561, 422–429 (Springer, 2014).
