# Supplementary material for: Discovering heuristics in a complex SAT solver with large language models
Source: Nat Commun. 2026 Jul 17;17:9008. doi: 10.1038/s41467-026-74949-2 (PMC13507263; doi:10.1038/s41467-026-74949-2)
Supplement: Supplementary file 2 [file 41467_2026_74949_MOESM2_ESM.pdf]

nature portfolio

Peer Review File

Discovering heuristics in a complex SAT solver with large language models

Corresponding Author: Professor Shaowei Cai

This file contains all reviewer reports in order by version, followed by all author rebuttals in order by version.

Version 0:

Reviewer comments:

Reviewer #1

(Remarks to the Author)
The paper presents AutoModSAT, a new satisfiability solver. AutoModSAT is created by mutating the code base of an existing SAT solver by replacing specific C++ functions with newly generated implementations generated by a large language model. The authors evaluate AutoModSAT against state-of-the-art satisfiability solvers on a large corpus of datasets with significant performance improvements.

Strengths:
- significant performance improvements compared to the state-of-the-art (relevance)
- new idea for discovering SAT solver heuristics (novelty)
- relevant research problem (relevance)
- replication package (verifiability)

Weaknesses:
- paper not self contained (presentation)
- unconventional and confusing structure (presentation)
- correctness not evaluated (soundness)
- base solver for mutation-based approach unknown (soundness)

Relevance
The paper tackles a highly relevant research problem and provides significant performance improvements compared to the state of the art.

Novelty
The paper presents a novel SAT solver and a novel approach for implementing SAT solvers based on discovering new heuristics with the a large language model.

Presentation
The paper suffers severe presentation issues.

The paper's structure is unconventional and hence confusing. For instance, the paper presents experimental results before introducing the new solver AutoModSAT. The "Discussion" sections appears to be a typical "Conclusion" section, discussing, framing, and summarizing the results of the entire paper, not just the experiments. An explicit "Conclusion" section is missing.

Crucially, the paper does not properly introduce the design of the new SAT solvers ModSAT and AutoModSAT. While there is a small "Methods" section, it remains too brief and technical to be understood without prior knowledge on the implementation details of SAT solving algorithms. In fact, a detailed methods section is part of the supplementary material. The supplementary material includes a missing "Background" section as well, explaining the necessary background knowledge on SAT solving algorithms.

It seems as if the 5000 word limits of the journal severely dismembered the submitted article. As is, the main article is not

self-contained and core contributions are instead moved to the not-anymore-supplementary material. In fact, the main article frequently refers to sections and figures in the supplementary material. The authors should consider submitting their article to a journal without a page or word restriction.

Furthermore, there are a range of typographical, spelling, and grammatical mistakes (see minor comments below).

Verifiability
The authors submitted a replication package of their work alongside the main paper, which is highly appreciated. Apart from that, the presentation issues mentioned above make it hard to judge verifiability. Given that the paper uses a large language model, the results might not be reproducible without access to that large language model (an inherent issue in modern AI research).

Soundness
There are a multitude of soundness issues.

First, and most crucially, the correctness of the proposed SAT solver is not evaluated. The authors present a new SAT solver in which code for certain heuristics was generated by an LLM but there is no evidence that the generated code does not impact the correctness of the mutated code base. Do all tested solvers report the same results (i.e., satisfiable vs. unsatisfiable) for all propositional formulas in the dataset?

Second, while the LLM-based approach mutates the code base of an existing solver, the authors do not discuss _which_ base implementation is mutated (i.e., which SAT solver serves as the baseline). Do the authors improve upon an existing state-of-the-art SAT solver? Do they implement a new, custom solver? Moreover, looking at the supplementary material, the LLM discovers significantly more complex heuristics, replacing a simple boolean expression with a large C++ function. How was the LLM able to come up with such a complex new heuristic? Is it part of the training data? Why was the initial expression so simple in comparison? How do the discovered heuristics compare to heuristics used in state-of-the-art solvers? A respective qualitative discussion remains very brief given the word limit.

Third, the main motivation behind the new approach for developing SAT solvers is unsound. L189 states that "The current architecture of complex solvers often have extensive functions, resulting in poor modularity and intricate data structures" (it seems that "functions" refer to C/C++ functions). Highly optimized state-of-the-art solvers require advanced decision procedures and "intricate" data structures necessarily. In fact, the author's answer to this issue is to use LLMs to generate yet again rather complex C++ functions.

Fourth, the proposed algorithm is heavily based on LLM use but it is never discussed which LLM is used and how it is trained.

Judgment:
While the paper presents a novel and promising solution to the satisfiability problem, the severe presentation and soundness issues mandate rejection.

Minor Comments
- The abbreviation "SOTA" in the abstract could be avoided given that it is used only once.
- L22: extra space before citation
- L22: "which also refer to as algorithm configuration" -> "also referred to as algorithm configuration"?
- L25: "Whereas, ..." is not a sentence.
- L27: "fascinating" -> Such subjective judgments should not be part of a scientific paper.
- L54: "compatabability" -> "compatibility"
- L55: missing space
- L83: "substaintial" -> substantial
- In Figure 2, it took me a while to realize that the first sentence of the caption refers only to the subfigure a, not Figure 2 as a whole.
- L107: "Owing to the superior performance of AutoModSAT when utilizing DeepSeek-V3, we only report results obtained from it in this paper." -> This sentence remained unclear. What does "it" refer to? Why should the reporting of results depend on performance?
- L108: Why is the "standard 5000 seconds timeout bound in the SAT competition" considered to be excessively long by the authors? The timeout was probably chosen for a good reason by the SAT competition. It seems that the timeout is only reduced by the authors for the training of the LLM but it is not clear. How would this timeout have impacted the training of the large language model?
- L127: "Overall, AutoModSAT establishes itself as a robust and efficient solver" -> What does robustness mean in this context? How does performance data give insight into robustness?
- The "Performance" subsection mixes presentation of results with discussions on experiment design, which is confusing.
- Figure 2a shows that there is at least one dataset where AutoModSAT does not perform best. This is not a problem but is there any reason why this dataset could be special in this regard?
- The beginning of the results section already makes claims of the effectiveness of AutoModSAT, such as "Overall, AutoModSAT can deliver superior performance without requiring additional instance-specific fine-tuning workload, which can significantly reduces both time and labor costs in real-world applications.", without any evidence for that claim. Such a statement is better put into the conclusion, once respective evidence is collected (e.g., via experiments).
- Regarding the selection of datasets, an unanswered question is why small dataset families are removed from the SAT

competition dataset. It might be that such smaller families contain large or challenging instances.

(Remarks on code availability)
I had only a short look at the code base. The mid-sized code base seems to be complete but lacks the documentation to foster reuse in future research or by practitioners. There is no README file and there is no source code documentation except for rare and brief code comments. The command-line interface does not provide any user help. There is no README file and there is no LICENSE.

Reviewer #2

(Remarks to the Author)
This work proposes a method called AutoModSAT that, through the use of LLMs for code generation, aims to outperform SOTA solvers by 30%. It also claims a 20% speedup on average over parameter-tuned alternatives of the SOTA solvers.

After a careful reading of your research, I have several points of concern:

1) Lack of information for reproducibility and viability. A scientific advancement cannot be considered complete if it is not reproducible. There is a lack of information on key hyperparameters such as temperature, and an absence of economic/computational cost details. How can we know if the results are not a product of luck or an experimental budget inaccessible to others? We know that in code generation, the choice of temperature affects the outcome of new heuristics. Furthermore, empirically, not all LLMs function the same at a given temperature, for example, 0.8.

The authors mention, "Owing to the superior performance of AutoModSAT when utilizing DeepSeek-V3, we only report results obtained from it in this paper. Due to the standard 5000 seconds timeout bound in the SAT competition is excessively long, we use half the median solving time reported from the original solvers as the timeout threshold in training, which can also ensure evaluation of the generalization capabilities." (lines 107-110). Where was DeepSeek-V3 executed, and with what hyperparameters? What other LLMs was it compared against? If they paid, how many tokens were used, and what was the economic cost? Without this information, it is very difficult to even approach a reproduction of your results.

2) The "Automation" is partial and depends on heavy human intervention. The system is presented as "Automatically discovering heuristics in a complex SAT solver," however, the complex SAT solver was created by you: ModSAT. That is your baseline, and it has a simplified code design. Furthermore, the role of human intervention is not entirely clear. It appears that the preparation of the code to prevent LLM errors was done by you, and a large part of the success lies there. This suggests the process is not fully automatic, but requires a human-in-the-loop cycle where an expert prepares the ground and corrects the environment to guide the LLM. While this is practical, it weakens the narrative of a fully autonomous system. The LLM operates in a 'protected' and simplified environment. And while there is nothing wrong with that, it would be appropriate to be transparent about it, mentioning the prior human work (building ModSAT and implementing error validations), and that the large 50% advantage is over your own creation (which naturally provides a wide margin of improvement). The true measure of success should be the final advantage over SOTA solvers, which, although notable, is less than 50%.

I suggest you refine your narrative so that these points are clear, to avoid creating false expectations.

3) Generalization is ignored, and there could be overfitting. The method specializes a solver for each dataset but does not demonstrate that the discovered solutions are not overfitted to those specific problems. Cross-validation tests are missing: how does the solver optimized for 'EDA' perform on the 'cryptography' dataset? Without this, we don't know if the LLM is discovering general principles or simply 'tricks' for particular instances. Even if they were tricks for particular instances, this wouldn't be wrong, but again, it is important to provide this type of information to take it into account when trying to reproduce your experiments.

4) Lack of ablation studies. Although the paper mentions the comparison of Prompt Optimization: Figure 6 of the supplementary material directly compares the system's performance using the "Original Prompt" versus the "Updated" (optimized) one. The results consistently show that the optimized prompt improves performance, thus isolating and demonstrating the value of that specific component. However, other experiments are missing:

4.1) The Presearch Strategy: There is no comparison showing the final performance of AutoModSAT with this strategy versus an approach without it (e.g., a full evolutionary search over all seven heuristics). Its benefit is argued theoretically in terms of efficiency but not demonstrated empirically.

4.2) The LLM Agents (Repairer and Evaluator): The impact of the "Repairer" and "Evaluator" agents is not quantified. We do not know how many errors the repairer corrected or how many redundant evaluations the evaluator avoided, so their real contribution to the success of the process is not measured.

4.3) The Modularization Principles: The need to create a modularized solver like ModSAT is presented as a fundamental premise but is not validated experimentally against an alternative approach. It would be interesting to justify why.

I am not saying you should run all these experiments, but my point is that you should be able to measure how your system behaves when parts of it are deactivated. Perhaps there are modules that do not contribute much, or some that carry all the

relevance.

5) Is it really discovering new heuristics? The authors make a strong claim by stating they are "unprecedented heuristic rules" (caption Figure 3), but it could be argued that the LLM is performing a clever recombination of known concepts rather than a truly from-scratch invention. Assessing fundamental 'novelty' is difficult, but the presentation could be more nuanced.

I have several doubts about your code:

1) The files executor_agent.py and assistant_agent.py show a system prompt (variable DEFAULT_SYSTEM_MESSAGE). How did you arrive at that one? Did you test other different system prompts? What were the results? This comes back to the issue of reproducibility. Prompts are very relevant and affect the quality of the response, especially the system prompt. There are no comments on this in the paper or the supplementary material.

2) In the llm_api.py file, I have some doubts. It appears:

llm_api = LocalCallAPI(api_base="http://172.26.1.16:31251/v1",
api_key="sk-",
model_name="modelscope/modelscope/Llama-2-70b-chat-ms")

Did you use llama-2-70b? If so, where? You are supposed to have used DeepSeek-V3. It also defaults to temperature=0.2, why? There is also a function for GPT (which I assume was discarded based on the paper's description, and it appears with temperature=0.5).

In the file main_ea_automodsat.py, there is:

parser.add_argument('--llm_model',
type=str,
default="deepseek-chat",
choices=["gpt-4o-2024-08-06", "gpt-4-1106-preview", "gpt-3.5-turbo", "deepseek-reasoner", "o1-2024-12-17", "deepseek-ai/DeepSeek-R1", "deepseek-chat"])

There are many LLMs here. In the end, you use deepseek-chat. Which month's version is that, is it V3? Why is deepseek-chat the default and not some of the other LLMs?
I suggest that the code should have a connection with what you write in the paper to avoid confusion. The decisions in the code affect the results.

Final comments to the authors:

I find your proposal and the obtained results interesting. All my doubts lie in how viable and reproducible it is. Therefore, information is missing, especially from the perspective of someone who uses LLMs daily, because the use of the LLM is the heart of your mechanism. In its current state, the paper uses LLMs almost as a "black box." This can be mitigated by making both the hyperparameters (and their selection process) and the number of tokens used in all experiments, along with the associated economic costs, transparent.

(Remarks on code availability)
I reviewed the code. A README file explaining how to run and compile the code needs to be added, as I see that it has an "examples\ModSAT\module_function\modsat\CMakeLists.txt" file.

It might be a good idea to add a docker (or similar) so that the entire system can be run in an encapsulated environment.

Version 1:

Reviewer comments:

Reviewer #2

(Remarks to the Author)
I appreciate the authors' efforts in addressing my previous comments. After carefully reviewing the revised manuscript and the accompanying responses, several statements would benefit from further clarification for the sake of transparency.

1) On the "SOTA" narrative: The paper presents its results as surpassing the state of the art in SAT solvers, when what it actually demonstrates is the automation of domain specialization. Each generated solver is structurally overfitted to its specific dataset (cryptography, EDA, scheduling, etc.) and lacks transferability---changing the instance distribution requires regenerating heuristics from scratch. This is a valid contribution to solver engineering, but fundamentally different from an advance in generalist solvers. The comparison with Kissat/CaDiCal, even with SMAC3, is asymmetric: it compares code synthesis (unlimited search space) against parameter configuration (space predefined by the original designer). The authors should reframe their contribution as "automation of niche solvers" rather than "surpassing state of the art." Additionally, no cross-dataset transfer experiments are reported, which would be the definitive test to distinguish legitimate domain specialization from structural overfitting.

2) On the generated code in Figure 3:

```
fast_avg = 0.9 * fast_avg + 0.1 * curr_fast;
if (ratio > 1.2)
conflict_scale = 0.8
```

The LLM is simply fuzzing magic constants. It has not discovered a new theory about conflict structure in Boolean logic. It has merely found that, for that dataset, a moving average with alpha=0.1 works better than 0.05. There is no understanding here---just stochastic parameter search disguised as code generation.

This should be made transparent, along with the risks of using LLMs for such tasks.

3) On Software Engineering Quality and Robustness: Beyond the theoretical concerns of the generated code, there is a critical engineering defect in Figure 3 that compromises the solver's viability. The LLM-generated restart_function utilizes static local variables (e.g., static double fast_avg, static int restart_count).

In C++, this creates shared state across all instances of the Solver class. This violates object-oriented encapsulation and renders the solver non-reentrant and non-thread-safe. If this solver were used in a modern parallel portfolio (standard in industrial SAT solving) or instantiated multiple times in a single process, the instances would corrupt each other's internal state. Accepting such code demonstrates a lack of rigorous validation of the structural correctness of the heuristics, validating only their performance on a sequential benchmark. This represents a clear regression in software engineering quality relative to the baseline solvers.

This limitation should be explicitly acknowledged, and the authors should discuss possible mitigation strategies. It is clear that such a system could not be used in industrial applications unless it undergoes a rigorous formal verification process.

4) On DeepSeek-V3 selection: The statement "We only report results obtained from DeepSeek-V3 due to its superior performance" should explicitly note that the reasoning is in supplementary material to avoid misunderstandings.

5) Other points

Statistical concerns:

- Results are means of only 3 runs without confidence intervals. Given the high variance in SAT solving, this is insufficient to support superiority claims.
- On Zamkeller, AutoModSAT loses significantly to Kissat-para (2053.21 vs 486.5), yet this is downplayed. The claim of "shortest PAR-2 in 8/11 datasets" obscures that some losses are substantial.

Conceptual issues:

- The "75% budget reduction" claim assumes function independence, which the authors themselves contradict when mentioning "complex dependencies."
- The claim of "first successful attempt" to optimize complex solvers with LLMs is questionable given AutoSAT (ref 19) from the same group. This is strange. Wasn't his previous work a successful attempt?
- The process requires human experts to prepare the modularized solver (lines 181-182). This qualifies the "automatic optimization" claim and should be reflected in the title/abstract.

The work is valuable; however, the claims would benefit from being more measured. In particular, clarifying the scope of AutoModSAT and explicitly discussing the risks of using LLMs for heuristic generation in this domain would strengthen the paper and improve its overall rigor.

(Remarks on code availability)

Reviewer #3

(Remarks to the Author)

The paper presents AutoModSAT, a modular SAT solver explicitly designed to be augmented by Large Language Models (LLMs) through automatic heuristic generation. The framework relies on a relatively complex pipeline involving multiple LLM agents, which are used to generate new heuristics, detect semantic similarity with existing code, evaluate performance, and repair incorrect implementations when necessary via an evolutionary algorithm. At the end of the heuristic discovery process, the best-performing heuristics are integrated into the solver and used to accelerate SAT solving on benchmark instances. The authors claim that this approach is able to outperform the current single-core

state-of-the-art SAT solvers, based on benchmarks from the 2023 and 2024 SAT Competition main track.

Strengths:
1. Automatic discovery of SAT-solving heuristics is a central challenge in reducing the wall-clock time required to solve hard combinatorial problems. The paper leverages the generative capabilities of LLMs to address a core and long-standing problem in SAT solving.

2. The approach is evaluated on 435 challenging benchmark instances, including problems from the SAT Competition 2023 and 2024, instances generated using Picat, and real-world problems arising from Electronic Design Automation (EDA) scenarios. This constitutes a well-balanced mix of academic benchmarks and practically relevant instances.

Weaknesses:
1. Many essential details are compressed or deferred to the supplementary material, making the paper difficult to follow.

2. The manuscript contains a non-negligible number of grammatical and stylistic errors. The paper should be carefully proofread to improve clarity and readability.

3. Figure 2 is difficult to read.
a. Subfigure (a) relies on a radar plot with overlapping semi-transparent areas and legends, which makes it hard to compare solver performance. Radar plots are notoriously difficult to read when densely populated; a different visualization should be considered.
b. In subfigure (b), I suggest replacing the labels "para" and "Auto" with "ModSAT" and "AutoModSAT", respectively. Additionally, the repeated "% improvement" labels on the axes should be avoided.
c. In subfigure (c), it is unclear whether the three experiments correspond to the same experimental setup for each problem class. There is a floating "experiments" legend and a "NaN" label above it. Moreover, the meaning of the dots on the curves is not explained, the sampling intervals appear uneven, and in some cases (typically the blue curve) the plot terminates earlier than the others.
The figure and its caption require substantial clarification.

4. Times for the heuristic discovery process are missing. Reporting the time required to generate, evaluate, and integrate heuristics is essential to assess the practicality of the approach. These results should be provided, at least in the supplementary material.

5. (line 109) "We only report results obtained from DeepSeek-V3 in this paper due to its superior performance in optimizing the SAT solver". An ablation study involving different LLMs is presented in the supplementary material, but it is not referenced here. To support such a claim, a clear reference to it is required. Otherwise, the statement should be rephrased to reflect practical constraints rather than superiority.

6. Figure 1 (the graphical abstract) requires significant improvement. The layout is cluttered: arrows overlap figures, some lines nearly touch where they should not, and parts of the diagram are difficult to read. Greater care should be taken in the visual presentation.

7. In some datasets (e.g., Zamkeller), the performance of AutoModSAT is substantially worse than competing solvers. The paper does not provide an explanation for these cases, which raises concerns about robustness.

8. The paper repeatedly mentions 30%+ speedups, but it is unclear how these numbers are derived. The source of these improvements should be made explicit, for example by adding a dedicated column in Table 2.

9. It would be helpful to include a direct comparison between heuristics obtained with and without the presearch strategy and automatic prompt optimization. Quantitative evidence (e.g., runtime reduction or search-tree size reduction) would better support the authors' claims.

Major concerns:
1. The paper does not discuss whether the discovered heuristics are already known in the SAT literature and hence if the LLM is genuinely generating new heuristics or simply recalling the ones saw during training.

2. There are no formal guarantees regarding the correctness of the generated heuristics. Since arbitrary code is produced, an LLM could generate a heuristic that introduces subtle bugs (e.g., compromising completeness), which might not be detected by the evaluation pipeline.

3. (line 188) "This is a transferable research paradigm also applicable to other kinds of complex solvers, and thus opens new avenues for solver optimization". The paper repeatedly emphasizes that strong performance requires explicit solver modularization, which is stated to be missing in current SAT solvers. It is therefore unclear how this paradigm would transfer to other complex solvers without substantial manual redesign.

4. Hardware specifications used for the experiments are completely missing. This information is essential for reproducibility.

5. The paper uses the term $(1 + \lambda)$ Evolutionary Algorithm without explanation. A brief description or an appropriate citation should be provided.

6. The authors claim that the process is automatic multiple times throught the paper, but in the discussion session they mention that a human in-the-loop is required. The authors should clarify this. From what I undesrstand, humans are involved in multiple steps of the process, particularly (but not only) in the preparation of the base solver. Are humans necessary in other parts of the process? Can you claim that the entire process is automatic then?

Notes:
1. Although the section ordering is somewhat unusual, it appears consistent with the style of other Nature Communications articles.
2. In Table 1, the total number of problem instances evaluated should be explicitly stated.
3. The overall narrative and tone of the paper require revision. Several claims are overstated or insufficiently supported. A more modest and scientifically cautious tone is recommended.
4. The paper should explicitly state that the solver is intended for the main (single-core) track of the SAT Competition, and that parallel SAT solving is out of scope.
5. The paper would benefit from a clearer positioning with respect to recent LLM-based SAT and algorithm-design approaches, explicitly stating what AutoModSAT does not attempt to solve.
7. Some key methodological components (e.g., presearch pruning and evolutionary refinement) are relegated to the supplementary material but are essential for understanding the method and should be summarized in the main text.
8. Make the code in Figure 3 highlighted. Please avoid using a screenshot of the code but show code via text.
9. In page 3, line 59, there is no definition for A.

(Remarks on code availability)
The code was browsed and was found to include a README file containing instructions for the experiments. The experiments were not reproduced. Based on an initial inspection, no unusual or concerning aspects were identified.

Reviewer #4

(Remarks to the Author)
I co-reviewed this manuscript with one of the reviewers who provided the listed reports. This is part of the Nature Communications initiative to facilitate training in peer review and to provide appropriate recognition for Early Career Researchers who co-review manuscripts.

(Remarks on code availability)

Version 2:

Reviewer comments:

Reviewer #2

(Remarks to the Author)
I thank the authors for following my suggestions. My main remaining criticism had been that the claims made in the introduction were not supported by the results and were somewhat overstated. With the revisions now made, the paper is more honest in its framing and can present the novelty and strength of the research more clearly.

(Remarks on code availability)

Reviewer #3

(Remarks to the Author)

The revised version addresses several of the reviewers' comments, and a number of improvements have been made. Overall, the work has merit, but the manuscript would benefit from a clearer structure, improved self-containment, and more careful attention to the presentation of figures.

The presentation of the manuscript still appears somewhat fragmented and at times difficult to follow. A substantial amount of essential information is deferred to the supplementary material. While this may partly be due to space constraints, the current organization forces the reader to frequently consult the supplementary sections for important methodological and experimental details.
The manuscript would benefit from a clearer restructuring that moves some of the most relevant material into the main text. For example, the performance analysis explaining why the method performs worse on Zamkeller, or the average time required to generate the heuristics.

Parts of the surrounding discussion also feel somewhat unfocused. A more streamlined and structured presentation of the methodological details would significantly improve readability. For instance, improving the prompt optimization procedure, the presearch strategy, and certain aspects of the experimental setup.

Finally, while some figures were revised, several of them would benefit from additional checks and greater care in their preparation to ensure clarity and consistency. For example, Figure 2, particularly in areas where labels overlap with the figure itself or where the use of similar colors in the radar plot makes interpretation difficult.

(Remarks on code availability)
The code was browsed and was found to include a README file containing instructions for the experiments. The experiments were not reproduced. Based on an initial inspection, no unusual or concerning aspects were identified.

Reviewer #4

(Remarks to the Author)
I co-reviewed this manuscript with one of the reviewers who provided the listed reports. This is part of the Nature Communications initiative to facilitate training in peer review and to provide appropriate recognition for Early Career Researchers who co-review manuscripts.

(Remarks on code availability)

Version 3:

Reviewer comments:

Reviewer #3

(Remarks to the Author)
The authors have addressed the main concerns raised in the previous review. The manuscript has been improved in terms of structure, clarity, and completeness, with relevant methodological and experimental details now included in the main text. The reorganization of the Method and Results sections and the revisions to the figures have also contributed to a more coherent and readable presentation.
The current version represents a satisfactory improvement over the previous submission.

(Remarks on code availability)
The code was browsed and was found to include a README file containing instructions for the experiments. The experiments were not reproduced. Based on an initial inspection, no unusual or concerning aspects were identified.

Reviewer #4

(Remarks to the Author)

I co-reviewed this manuscript with one of the reviewers who provided the listed reports. This is part of the Nature Communications initiative to facilitate training in peer review and to provide appropriate recognition for Early Career Researchers who co-review manuscripts.

(Remarks on code availability)
The authors have addressed the main concerns raised in the previous review. The manuscript has been improved in terms of structure, clarity, and completeness, with relevant methodological and experimental details now included in the main text. The reorganization of the Method and Results sections and the revisions to the figures have also contributed to a more coherent and readable presentation.
The current version represents a satisfactory improvement over the previous submission.

Open Access This Peer Review File is licensed under a Creative Commons Attribution 4.0 International License, which permits use, sharing, adaptation, distribution and reproduction in any medium or format, as long as you give appropriate credit to the original author(s) and the source, provide a link to the Creative Commons license, and indicate if changes were made.
In cases where reviewers are anonymous, credit should be given to 'Anonymous Referee' and the source.
The images or other third party material in this Peer Review File are included in the article’s Creative Commons license, unless indicated otherwise in a credit line to the material. If material is not included in the article’s Creative Commons license and your intended use is not permitted by statutory regulation or exceeds the permitted use, you will need to obtain permission directly from the copyright holder.
To view a copy of this license, visit https://creativecommons.org/licenses/by/4.0/

Response to reviewers

Dear Associate Editor and the Reviewers,

We would like to sincerely thank the reviewers for their valuable comments, which have greatly helped us improve the quality of this paper. To summarize, both reviewers recognized that our work has addressed a highly relevant problem via a novel approach, that is using large language models to discover heuristics for the SAT solvers.

The reviewers pointed out several issues regarding presentation, soundness, and reproducibility. We fully acknowledge that these issues are important and have made substantial revisions to address them. Specifically, we have moved essential background and methodological content from the Supplementary Materials into the main text to improve readability and ensure the paper is overall self-contained. In addition, we have added more detailed descriptions of our experiments, and have conducted new ablation studies. To enhance reproducibility, we have also updated the released code by adding a detailed README file and license file, and removing unnecessary redundant code.

The detailed comments are listed below, followed by our response and the resolution typed in red. Modified texts appear in red in the revised manuscript.

Best regards,
Authors

Reviewer Comments

Reviewer #1

Presentation The paper suffers from severe presentation issues:

- The paper's structure is unconventional and hence confusing. For instance, the paper presents experimental results before introducing the new solver AutoModSAT. The "Discussion" sections appears to be a typical "Conclusion" section, discussing, framing, and summarizing the results of the entire paper, not just the experiments. An explicit "Conclusion" section is missing.

  Thank you for the valuable suggestion. In fact, we attempted to follow the typical structure of a Nature Communications paper after reading quite a few published ones. That being said, we agree that the current "Discussion" section reads more like a conclusion. In the revision, we have updated the "Discussion" section and moved the existing content to a new "Conclusion" section.

- Crucially, the paper does not properly introduce the design of the new SAT solvers ModSAT and AutoModSAT. While there is a small "Methods" section, it remains too brief and technical to be understood without prior knowledge on the implementation details of SAT solving algorithms. In fact, a detailed methods section is part of the supplementary material. The supplementary material includes a missing "Background" section as well, explaining the necessary background knowledge on SAT solving algorithms.

  Thank you for the helpful suggestion. In the revision, we have added a brief introduction about SAT and an overview of ModSAT in the "Methods" section, to improve the readability and help readers

understand the problem more easily.

- It seems as if the 5000 word limits of the journal severely dismembered the submitted article. As is, the main article is not self-contained and core contributions are instead moved to the not-anymore-supplementary material. In fact, the main article frequently refers to sections and figures in the supplementary material. The authors should consider submitting their article to a journal without a page or word restriction.

  Thank you for this constructive suggestion. We think Nature Communications is appropriate for our paper since the topic fits its scope very well. The previous version contained approximately 4,000 words. In the revision, we have used the remaining 1,000 words to improve readability and integrate essential content from the supplement into the main text (including key descriptions and an important figure). We also reduced cross-referencing to the supplement and ensured that the main article now stands on its own. We hope these changes address your concern.

- Furthermore, there are a range of typographical, spelling, and grammatical mistakes (see minor comments below).

  Thank you for your detailed reading of this paper, and we have corrected all these mistakes, see the response in the "Minor Comments" part.

Soundness There are a multitude of soundness issues:

1. First, and most crucially, the correctness of the proposed SAT solver is not evaluated. The authors present a new SAT solver in which code for certain heuristics was generated by an LLM but there is no evidence that the generated code does not impact the correctness of the mutated code base. Do all tested solvers report the same results (i.e., satisfiable vs. unsatisfiable) for all propositional formulas in the dataset?

   Thank you for the thoughtful suggestion. We agree that ensuring solver correctness is essential. Since our modifications are confined to heuristics, we did not modify any soundness-critical components (e.g., CNF parsing, Boolean constraint propagation, backtracking, or proof logging). Therefore, in principle, the satisfiability should be unaffected by the LLM-generated code. Nonetheless, to preclude any possibility of error, we have added explicit SAT/UNSAT validation. For SAT cases, the solver outputs the satisfying assignment and checks the correctness by verifying the original CNF. For UNSAT cases, the solver outputs a DRAT UNSAT proof and is verified using drat-trim. For each dataset, we also compare the SAT/UNSAT status reported by all tested solvers (baseline solvers and AutoModSAT-discovered solvers). In our experiments, no inconsistencies in SAT/UNSAT status were observed across the solvers. More details can be found in Supplementary Section 6.7.

2. Second, while the LLM-based approach mutates the code base of an existing solver, the authors do not discuss _which_ base implementation is mutated (i.e., which SAT solver serves as the baseline). Do the authors improve upon an existing state-of-the-art SAT solver? Do they implement a new, custom solver? Moreover, looking at the supplementary material, the LLM discovers significantly more complex heuristics, replacing a simple boolean expression with a large C++ function. How was the LLM able to come up with such a complex new heuristic? Is it part of the training data? Why was the initial expression so simple in comparison? How do the discovered heuristics compare to heuristics used in state-of-the-art solvers? A respective qualitative discussion remains very brief given the word limit.

Thank you very much for your valuable feedback. As previously mentioned, we have added an overview of ModSAT in the "Methods" Section. The base solver ModSAT is based on MiniSat, enhanced with rephasing mechanisms, and then modularized through the three modification principles we proposed. We use MiniSat as our backbone because it is widely used and stable in industrial applications. And the rephasing heuristic was added because it significantly impacts solver performance and encapsulates substantial human expertise, making it particularly suitable for LLM-based adaptation and refinement. After modifying, ModSAT exhibits a similar performance as MiniSat (slightly better in some datasets, as shown in Table 2, because of dynamic restart and rephase mechanisms). Despite the similar performance, the code structure changes, and the modifications can be found in the code (primarily in the Solver.h and Solver.cc files under the modsat folder).

As for the question of whether the heuristics discovered by the LLM are part of its training data, actually we could not know the training procedure and data of LLMs (such as GPT and DeepSeek). Even though DeepSeek's model is open-source, the training data and pipeline code are not public. This is indeed a broad and open-ended question, and some papers have attempted to discuss this phenomenon. However, there is no universally accepted detailed answer yet. That being said, this question could be translated into another one: whether these discovered heuristics already exist. We have attempted to evaluate this by conducting some comparisons in the revision. From our evaluation, it seems that, compared with the original heuristics, the heuristics discovered by AutoModSAT tend to integrate multiple rules, resulting in more complex and hybrid heuristics. The details can be found in Supplementary Section 7.2.

3. Third, the main motivation behind the new approach for developing SAT solvers is unsound. L189 states that "The current architecture of complex solvers often have extensive functions, resulting in poor modularity and intricate data structures" (it seems that "functions" refer to C/C++ functions). Highly optimized state-of-the-art solvers require advanced decision procedures and "intricate" data structures necessarily. In fact, the author's answer to this issue is to use LLMs to generate yet again rather complex C++ functions.

   Thank you for the thoughtful comment, and apologies for the ambiguity in the writing. We agree that highly optimized, state-of-the-art SAT solvers necessarily rely on advanced decision procedures and intricate data structures. What we want to emphasize is that the current architecture of complex solvers is not well-modularized enough for LLMs to effectively optimize. We have revised the statement that causes ambiguity in line 226 of the revision.

4. Fourth, the proposed algorithm is heavily based on LLM use but it is never discussed which LLM is used and how it is trained.

   Thank you for the question. It is noted in the Experiment Setup that Deepseek-V3 is used. Actually, we do not train this model, but only perform inference using it. In addition, we have now provided the details of the parameters used in the LLM in Supplementary Section 6.4 of the revision.

Minor Comments

- The abbreviation "SOTA" in the abstract could be avoided given that it is used only once.

  We replace "SOTA" with "state-of-the-art" in the abstract.

- L22: Extra space before citation.

  The extra space is deleted in line 22.

- L22: “which also refer to as algorithm configuration” → “also referred to as algorithm configuration”.

  “which also refer” is changed to to “also referred” in line 23.

- L25: “Whereas, ...” is not a sentence.

  “Whereas” is changed to “However,” in line 25.

- L27: “fascinating” → Such subjective judgments should not be part of a scientific paper.

  “fascinating” is removed in line 27.

- L54: “compatabability” → “compatibility”.

  The typo is fixed in line 54.

- L55: Missing space.

  The space is added in line 55.

- L83: “substaintial” → “substantial”.

  The typo is fixed in line 84.

- In Figure 2, it took me a while to realize that the first sentence of the caption refers only to the subfigure a, not Figure 2 as a whole.

  Apologize for that, and we add “this subfigure” to make it clear in Figure 2.

- L107: “Owing to the superior performance of AutoModSAT when utilizing DeepSeek-V3, we only report results obtained from it in this paper.” -> This sentence remained unclear. What does “it” refer to? Why should the reporting of results depend on performance?

  “it” refers to DeepSeek-V3. We chose to report the main results with DeepSeek-V3 because it is effective and also less expensive. We have revised the sentence to avoid ambiguity in line 108. Moreover, a subsection is added in Supplementary Section 6.4 of the revision to compare the performance of different LLMs .

- L108: Why is the “standard 5000 seconds timeout bound in the SAT competition” considered to be excessively long by the authors? The timeout was probably chosen for a good reason by the SAT competition. It seems that the timeout is only reduced by the authors for the training of the LLM, but it is not clear. How would this timeout have impacted the training of the large language model?

  Thank you for the comment. To clarify, we did not train LLMs, but only use LLMs to discover heuristics in a solver. Our pipeline is an iterative loop: the LLM proposes a change, then we compile and run the modified solver, and then measure the performance. In this loop, the wall-clock cost is dominated by solver runs, not LLM queries.

  We agree that the 5000 second timeout is the standard in the SAT Competition, but it is expensive when used inside an inner optimization loop because any single hard instance forces a wait until timeout. Using 5000 seconds would make 50 iterations take about 3 days. Therefore, during the search phase we run at a shorter timeout to accelerate the feedback and reduce the search time. Despite this, for fairness and comparability, after the search we re-evaluate every resulting solver under the standard 5000-second timeout in the paper.

- L127: “Overall, AutoModSAT establishes itself as a robust and efficient solver” -> What does robustness mean in this context? How does performance data give insight into robustness?

  Thanks for pointing this out. We have deleted “robust” to avoid confusion in line 129.

- The “Performance” subsection mixes presentation of results with discussions on experiment design, which is confusing.

  Thank you for catching this. We have moved the discussions on experiment design to the “Experimental setup” part in line 113 to 115.

- Figure 2a shows that there is at least one dataset where AutoModSAT does not perform best. This is not a problem but is there any reason why this dataset could be special in this regard?

  Thank you for this insightful question. In fact, it is not very clear to us why the dataset is special, which is worth conducting a deep investigation and see whether the performance of AutoModSAT can be further enhanced for these dataset in the future.

- The beginning of the results section already makes claims of the effectiveness of AutoModSAT, such as “Overall, AutoModSAT can deliver superior performance without requiring additional instance-specific fine-tuning workload, which can significantly reduces both time and labor costs in real-world applications.”, without any evidence for that claim. Such a statement is better put into the conclusion, once respective evidence is collected (e.g., via experiments).

  We have removed this sentence from the beginning of the “Results” section.

- Regarding the selection of datasets, an unanswered question is why small dataset families are removed from the SAT competition dataset. It might be that such smaller families contain large or challenging instances.

  As demonstrated in our preliminary tests, very small families are susceptible to overfitting during the iterative optimization process, which can produce misleading improvements that fail to generalize and thus are not considered. We have added a note in line 83 to 84 of the revision.

Remarks on Code Availability I had only a short look at the code base. The mid-sized code base seems to be complete but lacks the documentation to foster reuse in future research or by practitioners. There is no README file and there is no source code documentation except for rare and brief code comments. The command-line interface does not provide any user help. There is no README file and there is no LICENSE.

Thank you for the helpful comment. The README and the LICENSE file have been added.

Reviewer #2

Main Concerns After a careful reading of your research, I have several points of concern:

1. Lack of information for reproducibility and viability. A scientific advancement cannot be considered complete if it is not reproducible. There is a lack of information on key hyperparameters such as temperature, and an absence of economic/computational cost details. How can we know if the results are not a product of luck or an experimental budget inaccessible to others? We know that in code generation, the choice of temperature affects the outcome of new heuristics. Furthermore, empirically, not all LLMs function the same at a given temperature, for example, 0.8.

   The authors mention, “Owing to the superior performance of AutoModSAT when utilizing DeepSeek-V3, we only report results obtained from it in this paper. Due to the standard 5000 seconds timeout bound in the SAT competition is excessively long, we use half the median solving time reported from the original solvers as the timeout threshold in training, which can also ensure evaluation of the generalization capabilities.” (lines 107-110). Where was DeepSeek-V3 executed, and with what

hyperparameters? What other LLMs was it compared against? If they paid, how many tokens were used, and what was the economic cost? Without this information, it is very difficult to even approach a reproduction of your results.

Thank you for raising the issue about reproducibility. In the revision, we have added a new subsection (Section 6.4 in Supplementary), which includes temperature settings and economic costs of different LLMs. Hopefully, it can address your concern of reproducibility and viability.

2. The "Automation" is partial and depends on heavy human intervention. The system is presented as "Automatically discovering heuristics in a complex SAT solver," however, the complex SAT solver was created by you: ModSAT. That is your baseline, and it has a simplified code design. Furthermore, the role of human intervention is not entirely clear. It appears that the preparation of the code to prevent LLM errors was done by you, and a large part of the success lies there. This suggests the process is not fully automatic, but requires a human-in-the-loop cycle where an expert prepares the ground and corrects the environment to guide the LLM. While this is practical, it weakens the narrative of a fully autonomous system. The LLM operates in a 'protected' and simplified environment. And while there is nothing wrong with that, it would be appropriate to be transparent about it, mentioning the prior human work (building ModSAT and implementing error validations), and that the large 50% advantage is over your own creation (which naturally provides a wide margin of improvement). The true measure of success should be the final advantage over SOTA solvers, which, although notable, is less than 50%.

   I suggest you refine your narrative so that these points are clear, to avoid creating false expectations.

   Thank you for pointing this out, and we agree that the entire process is not fully automatic. We have removed the term "automatic" in several places. Moreover, we have added a short remark in the "Discuss" section to clarify this.

3. Generalization is ignored, and there could be overfitting. The method specializes a solver for each dataset but does not demonstrate that the discovered solutions are not overfit to those specific problems. Cross-validation tests are missing: how does the solver optimized for 'EDA' perform on the 'cryptography' dataset? Without this, we don't know if the LLM is discovering general principles or simply 'tricks' for particular instances. Even if they were tricks for particular instances, this wouldn't be wrong, but again, it is important to provide this type of information to take it into account when trying to reproduce your experiments.

   Thank you for the suggestion and we have provided the cross-validation tests in Supplementary Section 6.6. It can be observed that overall the learned heuristics for a specific dataset do not generalize well to other datasets. This is reasonable since we leverage LLMs to generate SAT solvers that are customized for a specific class of instances and there might be a dramatic difference between different datasets.

4. Lack of ablation studies. Although the paper mentions the comparison of Prompt Optimization: Figure 6 of the supplementary material directly compares the system's performance using the "Original Prompt" versus the "Updated" (optimized) one. The results consistently show that the optimized prompt improves performance, thus isolating and demonstrating the value of that specific component. However, other experiments are missing:

   (a) The Presearch Strategy: There is no comparison showing the final performance of AutoModSAT with this strategy versus an approach without it (e.g., a full evolutionary search over all seven heuristics). Its benefit is argued theoretically in terms of efficiency but not demonstrated empirically.

(b) The LLM Agents (Repairer and Evaluator): The impact of the "Repairer" and "Evaluator" agents is not quantified. We do not know how many errors the repairer corrected or how many redundant evaluations the evaluator avoided, so their real contribution to the success of the process is not measured.

(c) The Modularization Principles: The need to create a modularized solver like ModSAT is presented as a fundamental premise, but is not validated experimentally against an alternative approach. It would be interesting to justify why.

I am not saying you should run all these experiments, but my point is that you should be able to measure how your system behaves when parts of it are deactivated. Perhaps there are modules that do not contribute much, or some that carry all the relevance.

Thank you for this valuable suggestion. We agree that systematic ablations are essential. In the revised manuscript and Supplementary Sections 6.4 and 6.5, we provide the following ablation studies in addition to those on the prompt optimization.

- Presearch Strategy: We compare AutoModSAT with the presearch strategy against a full evolutionary search over all seven heuristics under equal computation budgets, see Supplementary Table 7, which shows a clear efficiency gain of the presearch strategy.
- LLM Agents (Repairer and Evaluator): The ratios of errors corrected by the Repairer and the redundant candidates pruned by the Evaluator are reported in Supplementary Table 6.
- Modularization Principles: Supplementary Table 7 also includes the results for AutoModSat without modularization, which clearly show that AutoModSAT based on the modularized solver can achieve much better performance.

5. Is it really discovering new heuristics? The authors make a strong claim by stating they are "unprecedented heuristic rules" (caption Figure 3), but it could be argued that the LLM is performing a clever recombination of known concepts rather than a truly from-scratch invention. Assessing fundamental 'novelty' is difficult, but the presentation could be more nuanced.

   Thank you for this thoughtful comment. It is true that assessing the fundamental "novelty" is interesting but intrinsically hard, as exhaustively enumerating all prior heuristic implementations is infeasible.

   That being said, we attempt to evaluate this by conducting some comparisons. From our evaluation, it seems that, compared with the original heuristics, the heuristics discovered by AutoModSAT tend to integrate multiple rules, resulting in more complex and hybrid heuristics. More details can be found in Section 7.2 of the revised supplementary.

6. I have several doubts about your code:

   (a) The files executor_agent.py and assistant_agent.py show a system prompt (variable DEFAULT_SYSTEM_MESSAGE). How did you arrive at that one? Did you test other different system prompts? What were the results? This comes back to the issue of reproducibility. Prompts are very relevant and affect the quality of the response, especially the system prompt. There are no comments on this in the paper or the supplementary material.

   Thank you very much for reading our code so carefully, what you observed indeed reflects the history of our development. The DEFAULT_SYSTEM_MESSAGE in executor_agent.py and assistant_agent.py is legacy code from an early phase when we briefly experimented with locally deployed LLMs. We ultimately did not use that path in our experiments. Because of cost considerations (self-deployment required substantial GPU resources and our usage didnt demand that), we switched to query models via their API keys.

In the final version we did not use a custom system prompt. We relied on their default system prompt and standard settings. We therefore did not run a systematic comparison of alternative system prompts for the reported results. From a reproducibility standpoint, this choice improves generality and makes replication more straightforward: anyone using the same provider, model/version, and decoding parameters should be able to reproduce our outputs without needing any complex system prompt engineering.

We apologize for not clarifying this in the paper/supplementary and for leaving the legacy variable in the repository. We will (i) remove or clearly comment those lines in the code, and (ii) add a note in Supplementary 6.4 that our experiments used provider defaults (including model names/versions and decoding parameters) rather than a handcrafted system prompt.

(b) In the llm_api.py file, I have some doubts. It appears:

```
llm_api = LocalCallAPI(
api_base="http://172.26.1.16:31251/v1",
api_key="sk-",
model_name="modelscope/modelscope/Llama-2-70b-chat-ms" )
```

Did you use llama-2-70b? If so, where? You are supposed to have used DeepSeek-V3. It also defaults to temperature=0.2, why? There is also a function for GPT (which I assume was discarded based on the paper's description, and it appears with temperature=0.5).

In the file main_ea_automodsat.py, there is:

```
parser.add_argument('–llm_model', type=str, default="deepseek-chat", choices=["gpt-4o-2024-08-06", "gpt-4-1106-preview", "gpt-3.5-turbo", "deepseek-reasoner", "o1-2024-12-17", "deepseek-ai/DeepSeek-R1", "deepseek-chat"])
```

There are many LLMs here. In the end, you use deepseek-chat. Which month's version is that, is it V3? Why is DeepSeek-Chat the default and not some of the other LLMs? I suggest that the code should have a connection with what you write in the paper to avoid confusion. The decisions in the code affect the results.

These code is also the legacy code. In the early prototype version we tried several models (e.g., Qwen2.5, GPT-4o, LLAMA-3.3) and finally used DeepSeek-V3, but we did not fully prune the legacy code paths. Your point also connects directly to your Main Concern 1 about reproducibility and viability, and we add a Supplementary Section 6.4 to discuss it, and update the repository (README + configs) to show the precise model, version, and parameters used in our experiments.

Final comments to the authors I find your proposal and the obtained results interesting. All my doubts lie in how viable and reproducible it is. Therefore, information is missing, especially from the perspective of someone who uses LLMs daily, because the use of the LLM is the heart of your mechanism. In its current state, the paper uses LLMs almost as a "black box." This can be mitigated by making both the hyperparameters (and their selection process) and the number of tokens used in all experiments, along with the associated economic costs, transparent.

Thank you very much for your thoughtful and constructive comments. We fully agree that transparency and reproducibility are crucial, especially given that LLMs are central to our method. In response, we have added detailed information on the LLM hyperparameters, their selection process, the number of tokens used in all experiments, and the corresponding computational and economic costs in Supplementary Sections 6.4, 6.5, and 6.6. We have also updated the README file in our released code to include implementation details necessary for reproducing our results. We hope these updates address your concerns.

Remarks on Code Availability I reviewed the code. A README file explaining how to run and compile the code needs to be added, as I see that it has an "`examples\ModSAT\module\_function\modsat\CMakeLists.txt`" file.

It might be a good idea to add a docker (or similar) so that the entire system can be run in an encapsulated environment.

Thank you for the helpful suggestion. A detailed README file has been added. In fact we initially explored the option of setting up an isolated Docker system for the solvers. However, our experiments are highly dependent on the stability of CPU execution time. Introducing a virtualization layer (such as Docker) would add overhead and variability to the CPU runtime evaluation. Therefore, we ultimately decided not to use this method.

Response to reviewers

Dear Associate Editor and the Reviewers,

We would like to sincerely thank the reviewers for their valuable comments, which have greatly helped us improve the quality of this paper. Overall, all reviewers recognized that our work addresses an important problem with a novel approach, namely using large language models to discover heuristics for SAT solvers.

The reviewers also pointed out several issues, including the need for more comprehensive experiments, clearer presentation of figures and tables, and more precise language. We fully acknowledge these comments and have made substantial revisions accordingly.

Specifically, we have made the claims more accurate and concise by revising the very subjective statements in the main text, as well as removing those repetitive statements. The entire paper is also further polished, with the major text editings marked red. The title of the paper is slightly revised, as suggested by the reviewer. We also moved more details from the supplementary materials to the main paper. In addition, we have conducted more experiments to justify and strengthen our results.

Again, the detailed comments are listed below, followed by our detailed responses.

Best regards,
Authors

Reviewer Comments

Reviewer #2

main concern:

- On the "SOTA" narrative: The paper presents its results as surpassing the state of the art in SAT solvers, when what it actually demonstrates is the automation of domain specialization. Each generated solver is structurally overfitted to its specific dataset (cryptography, EDA, scheduling, etc.) and lacks transferability—changing the instance distribution requires regenerating heuristics from scratch. This is a valid contribution to solver engineering, but fundamentally different from an advance in generalist solvers. The comparison with Kissat/CaDiCal, even with SMAC3, is asymmetric: it compares code synthesis (unlimited search space) against parameter configuration (space predefined by the original designer). The authors should reframe their contribution as "automation of niche solvers" rather than "surpassing state of the art." Additionally, no cross-dataset transfer experiments are reported, which would be the definitive test to distinguish legitimate domain specialization from structural overfitting.

  Thank you for the detailed review. We agree that our contribution is indeed about the automation of niche solvers. We have emphasized that AutoModSAT is an LLM-driven optimization framework for domain-specific solvers in for example the Results and Conclusion sections. In particular, we have deleted "surpassing state of the art" in Conclusion and instead used "automation of niche solvers" for AutoModSAT. Thank you for pointing out this phrase to us.

Thank you for your suggestion on the cross-dataset test to see whether the solvers produced by AutoModSAT can generalize well to test data from the same domain. To this end, we have conducted the generalization tests based on three new sets of data from the SAT Competitions and another two newly-generated sets of data by Picat (Zamkeller is excluded since even for the training data AutoModSAT solver is not as competitive as "Kissat para"). The results are presented in Supplementary Section 4.6, from which it can be seen that the AutoModSAT solvers generalize well to the test data in the same domain.

- (*) On the generated code in Figure 3:
  fast_avg = 0.9 * fast_avg + 0.1 * curr_fast;
  if (ratio > 1.2) conflict_scale = 0.8

  The LLM is simply fuzzing magic constants. It has not discovered a new theory about conflict structure in Boolean logic. It has merely found that, for that dataset, a moving average with alpha=0.1 works better than 0.05. There is no understanding here—just stochastic parameter search disguised as code generation. This should be made transparent, along with the risks of using LLMs for such tasks.

  Thank you for this valuable comment. We would like to note that, in addition to the constants, the heuristics produced by LLMs indeed introduce an adaptive backtracking/rollback policy conditioned on the fast/slow signal ratio rather than a static one used in the original heuristics. More precisely, the new heuristics compute sliding-window (moving-average) estimates for fast_lbd and slow_lbd, and then use their ratio to select different regimes and trigger a decision-level rollback mechanism (approximately decision_level / 2) when the ratio indicates potential conflict escalation.

  On the other hand, we do agree that one should be careful about the constants. It is well known LLMs tend to be "vibe coding": A style of programming where LLMs iterate quickly by following intuition and feedback rather than a fully clear and transparent design. Therefore, after a few iterations the resulting programs may be empirically strong while lacking interpretability. We have pointed this out in the section "Analysis of discovered heuristics".

- (*) On Software Engineering Quality and Robustness: Beyond the theoretical concerns of the generated code, there is a critical engineering defect in Figure 3 that compromises the solver's viability. The LLM-generated restart_function utilizes static local variables (e.g., static double fast_avg, static int restart_count).

  In C++, this creates shared state across all instances of the Solver class. This violates object-oriented encapsulation and renders the solver non-reentrant and non-thread-safe. If this solver were used in a modern parallel portfolio (standard in industrial SAT solving) or instantiated multiple times in a single process, the instances would corrupt each other's internal state. Accepting such code demonstrates a lack of rigorous validation of the structural correctness of the heuristics, validating only their performance on a sequential benchmark. This represents a clear regression in software engineering quality relative to the baseline solvers.

  This limitation should be explicitly acknowledged, and the authors should discuss possible mitigation strategies. It is clear that such a system could not be used in industrial applications unless it undergoes a rigorous formal verification process.

  Thank you for catching this issue which has not been noticed previously. We have explicitly acknowledged it also in the section "Analysis of discovered heuristics". Inspired by your comment, a potential solution is instantiating the solver multiple times in a single process and record the standard statistics (e.g., conflicts, starts, decisions, propagations) of each instantiation and see whether they are identical.

If not, we can trace back where the problem arises manually or by LLMs. We have tested this procedure using LLMs, which can identify the issue about the static variables. In addition, we may also point out this issue to LLMs beforehand in the prompt for generating the codes.

- On DeepSeek-V3 selection: The statement "We only report results obtained from DeepSeek-V3 due to its superior performance" should explicitly note that the reasoning is in supplementary material to avoid misunderstandings.

  Thank you for the suggestion. We have added the reference to the supplementary material.

Statistical concerns:

- Results are means of only 3 runs without confidence intervals. Given the high variance in SAT solving, this is insufficient to support superiority claims in Table 2.

  Thank you for this helpful comment. We have updated the main results in Table 2 based on the 10 independent runs and included the mean as well as a 95% confidence interval to better support the claims. In fact, it is interesting to see that AutoModSAT overall possesses a lower variance than the others.

- On Zamkeller, AutoModSAT loses significantly to Kissat-para (2053.21 vs 486.5), yet this is downplayed. The claim of "shortest PAR-2 in 8/11 datasets" obscures that some losses are substantial.

  Thank you for pointing this out. In the revision, we have clearly pointed out that the performance of AutoModSAT is substantially worse than that of “Kissat para”. Moreover, we have also investigated the huge improvement from Kissat to “Kissat para” on Zamkeller in Supplementary Section 5.3. It can be found out that the strong performance of “Kissat para” on Zamkeller relies primarily on disabling two preprocessing stages in Kissat (variable elimination and formula simplification). More precisely, a one-factor ablation and a two-factor combination study show that setting `eliminate=0` and `simplify=0` explains the majority of the performance gain. It seems this aggressive preprocessing is detrimental for Zamkeller. In contrast, the current version of AutoModSAT only searches over 7 functions and does not control top-level preprocessing switches such as `eliminate` and `simplify`. Note that if we disable these two parameters manually, the performance of AutoModSAT can indeed be further improved on Zamkeller. However, it is still far from competitive compared with “Kissat para” since the baseline solver ModSAT is relatively weak on this dataset.

Conceptual issues:

- The "75% budget reduction" claim assumes function independence, which the authors themselves contradict when mentioning “complex dependencies.”

  Thank you for pointing this out. We have revised this statement to a more qualitative one “the pre-search can improve the computational efficiency of AutoModSAT”, and provides the empirical support in Supplementary Figure 8.

- The claim of "first successful attempt" to optimize complex solvers with LLMs is questionable given AutoSAT (ref 19) from the same group. This is strange. Wasn’t his previous work a successful attempt?

  Thank you for pointing this out. We have removed this statement in the revision. Compared to AutoSAT, AutoModSAT can achieve much superior performance by introducing several new components

such as an LLM-friendly modularized solver design, automatic prompt optimization to increase code-generation diversity, and a presearch strategy to narrow the effective search space.

- The process requires human experts to prepare the modularized solver (lines 181-182). This qualifies the "automatic optimization" claim and should be reflected in the title/abstract.

  Thank you for this helpful suggestion. As suggested, we have removed “automatically” in the title and the other relevant places have also been properly rephrased.

Reviewer #3

Weaknesses:

- Many essential details are compressed or deferred to the supplementary material, making the paper difficult to follow.

  Thank you for the comment, and we have moved the essential details about automatic prompt optimization and presearch into the main text.

- The manuscript contains a non-negligible number of grammatical and stylistic errors. The paper should be carefully proofread to improve clarity and readability.

  Thank you for your suggestion. We have thoroughly proofread the entire manuscript to improve the clarity and readability of the paper. Major revisions have been marked red.

- Figure 2 is difficult to read.

  - (a) Subfigure (a) relies on a radar plot with overlapping semi-transparent areas and legends, which makes it hard to compare solver performance. Radar plots are notoriously difficult to read when densely populated; a different visualization should be considered.
  - (b) In subfigure (b), I suggest replacing the labels “para” and “Auto” with “ModSAT” and “Auto-ModSAT”, respectively. Additionally, the repeated “% improvement” labels on the axes should be avoided.
  - (c) In subfigure (c), it is unclear whether the three experiments correspond to the same experimental setup for each problem class. There is a floating “experiments” legend and a “NaN” label above it. Moreover, the meaning of the dots on the curves is not explained, the sampling intervals appear uneven, and in some cases (typically the blue curve) the plot terminates earlier than the others.

  The figure and its caption require substantial clarification.

  Thank you for catching these issues. We have substantially revised Figure 2 and its caption as follows.

  - We have split the radar plot into three ones via proper group.
  - We have modified the labels in subfigure (b).
  - For subfigure (c), the three curves correspond to three different random seeds under the same experimental setup. We explicitly stated this in both the legend and the caption of the figure. All the other issues have been fixed. Note that the subfigure (c) is now separated out and presented as an individual figure (i.e. Figure 3) in the revision.

- Times for the heuristic discovery process are missing. Reporting the time required to generate, evaluate, and integrate heuristics is essential to assess the practicality of the approach. These results should be provided, at least in the supplementary material.

  Thank you for this helpful suggestion. We have added the time for the LLM coder, evaluator, and repairer agents to the Supplementary Section 4.4 Table 6 (since this part of time is overall similar across different dataset, we only present the ones for EDA). Overall, the single call time of each agent is typically around $\sim$20 s.

- (line 109) "We only report results obtained from DeepSeek-V3 in this paper due to its superior performance in optimizing the SAT solver". An ablation study involving different LLMs is presented in the supplementary material, but it is not referenced here. To support such a claim, a clear reference to it is required. Otherwise, the statement should be rephrased to reflect practical constraints rather than superiority.

  Thank you for the suggestion, and we have made a clear reference in "Experimental setup". Additionally, we replaced the term "due to its superiority performance" with "due to its competitive performance and meanwhile the low cost".

- Figure 1 (the graphical abstract) requires significant improvement. The layout is cluttered: arrows overlap figures, some lines nearly touch where they should not, and parts of the diagram are difficult to read. Greater care should be taken in the visual presentation.

  Thank you for the helpful comment. We have revised Figure 1 by adjusting the arrows and lines, as well as trimmed the figure to make it clearer and more concise.

- In some datasets (e.g., Zamkeller), the performance of AutoModSAT is substantially worse than competing solvers. The paper does not provide an explanation for these cases, which raises concerns about robustness.

  Thank you for the insightful comment. In the revised version, we have investigated the huge improvement from Kissat to "Kissat para" on Zamkeller in Supplementary Section 5.3. It can be found out that the strong performance of "Kissat para" on Zamkeller relies primarily on *disabling* two preprocessing stages in Kissat (variable elimination and formula simplification). More precisely, a one-factor ablation and a two-factor combination study show that setting `eliminate=0` and `simplify=0` explains the majority of the performance gain. It seems this aggressive preprocessing is detrimental for Zamkeller. In contrast, the current version of AutoModSAT only searches over 7 functions and does not control top-level preprocessing switches such as `eliminate` and `simplify`. Note that if we disable these two parameters manually, the performance of AutoModSAT can indeed be further improved on Zamkeller. However, it is still far from competitive compared with "Kissat para" since the baseline solver ModSAT is relatively weak on this dataset.

- The paper repeatedly mentions 30%+ speedups, but it is unclear how these numbers are derived. The source of these improvements should be made explicit, for example by adding a dedicated column in Table 2.

  Thank you for pointing this out. The speedup metric is defined at the end of "Experimental setup" in the Results section: speedup $= \frac{v_a - v_b}{\max(v_a, v_b)}$, where v_a is the PAR-2 of method A, v_b is the PAR-2 of method B.

- It would be helpful to include a direct comparison between heuristics obtained with and without the presearch strategy and automatic prompt optimization. Quantitative evidence (e.g., runtime reduction or search-tree size reduction) would better support the authors' claims.

Thank you for the suggestion. The ablation studies are provided in Supplementary Section 4.5.

Major concerns:

- The paper does not discuss whether the discovered heuristics are already known in the SAT literature and hence if the LLM is genuinely generating new heuristics or simply recalling the ones saw during training.

  Thank you for raising this point. There is a discussion in Supplementary Section 5.2 about this concern. The results show that compared with the original hand-crafted heuristics, the heuristics discovered by AutoModSAT tend to integrate multiple rules into more complex, hybrid decision policies, rather than reproducing a single known heuristic. Even though in a few instances, certain sub-rules are inspired by techniques used in prior SAT solvers, there are several key elements and combinations in the discovered heuristics that do not appear in existing solver implementations within our examined corpus.

- There are no formal guarantees regarding the correctness of the generated heuristics. Since arbitrary code is produced, an LLM could generate a heuristic that introduces subtle bugs (e.g., compromising completeness), which might not be detected by the evaluation pipeline.

  Thank you for your suggestion. We have added an explicit correctness validation pipeline for all generated solvers in Supplementary Section 4.7. Concretely, for every SAT result we independently check the returned assignment by substituting it into the original CNF to check whether every clause is satisfied. Any failing instance is flagged and the corresponding solver variant is rejected. For every UNSAT result we require a DRAT proof and verify it using the external checker drat-trim; any rejected proof invalidates the UNSAT claim and the solver is rejected.

- (line 188) "This is a transferable research paradigm also applicable to other kinds of complex solvers, and thus opens new avenues for solver optimization". The paper repeatedly emphasizes that strong performance requires explicit solver modularization, which is stated to be missing in current SAT solvers. It is therefore unclear how this paradigm would transfer to other complex solvers without substantial manual redesign.

  Thank you for this comment. We agree that our pipeline is not drop-in transferable to a new solver without additional work, especially since the current approach relies on an explicitly modularized, LLM-friendly architecture. Our intention was to highlight the methodological contributions in AutoModSAT, for example the three-agent loop, presearch strategy, and diversity-oriented prompt optimization. Despite this, we acknowledge that the original wording was too strong, and we have revised the statements in Discussion and Conclusion substantially to avoid overclaim.

- Hardware specifications used for the experiments are completely missing. This information is essential for reproducibility.

  Thank you for the suggestion. We have added the missing hardware specifications to Experimental setup in the Results section for reproducibility. Specifically, we compile all solvers with g++ 9.4.0, and run experiments on five identical servers. Each server is equipped with two AMD EPYC 7763 CPUs (64 cores per CPU, 128 physical cores per server) with a base frequency of 2.45 GHz. Unless otherwise stated, we run 100 solver processes in parallel during benchmarking.

- The paper uses the term $(1 + \lambda)$ Evolutionary Algorithm without explanation. A brief description or an appropriate citation should be provided.

Thanks for pointing out the missing information. The brief description and an appropriate citation are now provided in Presearch strategy of the Method Section.

- The authors claim that the process is automatic multiple times through the paper, but in the discussion session they mention that a human in-the-loop is required. The authors should clarify this. From what I understand, humans are involved in multiple steps of the process, particularly (but not only) in the preparation of the base solver. Are humans necessary in other parts of the process? Can you claim that the entire process is automatic then?

  Thank you for pointing this out. We agree that our pipeline still requires human-in-the-loop involvement, and we have clarified this in the revised manuscript and qualified any fully automatic claims. In particular, human expertise is primarily needed during the preparation of the base solver and correctness verification. As suggested by the other reviewer, we have removed "automatically" in the title and the other relevant places have also been properly rephrased.

Notes:

- Although the section ordering is somewhat unusual, it appears consistent with the style of other Nature Communications articles.

  Thank you for the positive note.

- In Table 1, the total number of problem instances evaluated should be explicitly stated.

  Thank you for the suggestion. In Table 1, the column previously labeled "size" indeed corresponds to the total number of instances evaluated. We have renamed it to "total number of instances" to avoid confusion.

- The overall narrative and tone of the paper require revision. Several claims are overstated or insufficiently supported. A more modest and scientifically cautious tone is recommended.

  Thank you for the recommendation. We have revised the manuscript to adopt a more cautious scientific tone, removing or rephrasing statements that could be interpreted as overclaims.

- The paper should explicitly state that the solver is intended for the main(single-core) track of the SAT Competition, and that parallel SAT solving is out of scope.

  Thank you for your suggestion. We have clarified in the main text that AutoModSAT targets the single-core setting in Baselines of the Results section.

- The paper would benefit from a clearer positioning with respect to recent LLM-based SAT and algorithm-design approaches, explicitly stating what AutoModSAT does not attempt to solve.

  Thank you for the suggestion. A discussion about the positioning of AutoModSAT has been added in the Discussion section.

- Some key methodological components (e.g., presearch pruning and evolutionary refinement) are relegated to the supplementary material but are essential for understanding the method and should be summarized in the main text.

  Thank you for the suggestion and this has been done.

- Make the code in Figure 3 highlighted. Please avoid using a screenshot of the code but show code via text.

  Thank you for your suggestion. We have updated Figure 3 (Figure 4 now) to present the code as typeset text with proper highlighting rather than a screenshot.

- In page 3, line 59, there is no definition for A.

  Thank you for catching this issue, the definition of A has been added.

Response to reviewers

Dear Associate Editor and the Reviewers,

We would like to thank the reviewers again for their valuable comments, which have greatly helped us improve the quality of this paper.

As before, reviewers' comments are first listed, followed by our responses in red. All modified text also appears in red in the revised manuscript.

Best regards,
Authors

Reviewer Comments

Reviewer #3

- The presentation of the manuscript still appears somewhat fragmented and at times difficult to follow. A substantial amount of essential information is deferred to the supplementary material. While this may partly be due to space constraints, the current organization forces the reader to frequently consult the supplementary sections for important methodological and experimental details. The manuscript would benefit from a clearer restructuring that moves some of the most relevant material into the main text. For example, the performance analysis explaining why the method performs worse on Zamkeller, or the average time required to generate the heuristics. Parts of the surrounding discussion also feel somewhat unfocused. A more streamlined and structured presentation of the methodological details would significantly improve readability. For instance, improving the prompt optimization procedure, the presearch strategy, and certain aspects of the experimental setup.

  Thank you for your valuable suggestions. We have substantially revised the manuscript as suggested.

  Firstly, all the methodological details have been moved from the supplement to the main text, especially those about ModSAT. The average time required to generate heuristics as well as the performance analysis explaining why the method performs worse on Zamkeller are also included in the main text.

  Secondly, we have restructured the Method section as follows: brief introduction to SAT, ModSAT, three principles behind ModSAT, presearch strategy, prompt optimization, and heuristic discovery, so that the presentation is more logical.

  Thirdly, we have also improved the presentation of the prompt optimization, the presearch strategy, and the experimental setup.

- Finally, while some figures were revised, several of them would benefit from additional checks and greater care in their preparation to ensure clarity and consistency. For example, Figure 2, particularly in areas where labels overlap with the figure itself or where the use of similar colors in the radar plot makes interpretation difficult.

  Thank you for this helpful suggestion. We have carefully rechecked all figures and revised them to improve clarity and consistency. In particular, we adjusted the layout of Figure 2 to avoid overlapping and used contrasting colors for different methods to enhance readability.

Response to reviewers

Dear Associate Editor and the Reviewers,

We would like to thank the reviewers again for their valuable comments and positive assessment of our revised manuscript. We are pleased that the reviewers found the revised version improved in structure, clarity, completeness, and presentation.

As before, reviewers' comments are first listed, followed by our responses in red. Since no further scientific or technical revisions were requested, we provide brief point-by-point responses below. We have also carefully addressed the editorial and production-stage requirements in the revised manuscript files and the completed author checklist.

Best regards,
Authors

Reviewer Comments

Reviewer #3

- The authors have addressed the main concerns raised in the previous review. The manuscript has been improved in terms of structure, clarity, and completeness, with relevant methodological and experimental details now included in the main text. The reorganization of the Method and Results sections and the revisions to the figures have also contributed to a more coherent and readable presentation. The current version represents a satisfactory improvement over the previous submission.

  We sincerely thank the reviewer for the positive assessment of our revised manuscript. We are pleased that the reviewer found that the manuscript has improved in terms of structure, clarity, and completeness, and that the methodological and experimental details are now adequately included in the main text.

- **Remarks on code availability:** The code was browsed and was found to include a README file containing instructions for the experiments. The experiments were not reproduced. Based on an initial inspection, no unusual or concerning aspects were identified.

  We thank the reviewer for inspecting the code repository and the README file. We are pleased that no unusual or concerning aspects were identified during the initial inspection. We have retained the README file and the experimental instructions in the repository, and we have also ensured that the Code Availability statement points to the repository and the version of the code used in this study.

Reviewer #4

- I co-reviewed this manuscript with one of the reviewers who provided the listed reports. This is part of the Nature Communications initiative to facilitate training in peer review and to provide appropriate recognition for Early Career Researchers who co-review manuscripts.

  We thank the reviewer and the co-reviewer for their assessment of our manuscript.

- **Remarks on code availability:** The authors have addressed the main concerns raised in the previous review. The manuscript has been improved in terms of structure, clarity, and completeness, with relevant methodological and experimental details now included in the main text. The reorganization of the Method and Results sections and the revisions to the figures have also contributed to a more coherent and readable presentation. The current version represents a satisfactory improvement over the previous submission.

  We sincerely thank the reviewer for the positive assessment. We are pleased that the reviewer found the revised manuscript to be improved in structure, clarity, and completeness, and that the relevant methodological and experimental details are now included in the main text.
